# Induction of GNMT by 1,2,3,4,6-penta-O-galloyl-beta-D-glucopyranoside through proteasome-independent MYC downregulation in hepatocellular carcinoma

**DOI:** 10.1038/s41598-018-37292-1

**Published:** 2019-02-13

**Authors:** Rajni Kant, Chia-Hung Yen, Jung-Hsien Hung, Chung-Kuang Lu, Chien-Yi Tung, Pei-Ching Chang, Yueh-Hao Chen, Yu-Chang Tyan, Yi-Ming Arthur Chen

**Affiliations:** 1 Center for Infectious Disease and Cancer Research (CICAR), Kaohsiung Medical University, Kaohsiung, Taiwan; 2 Department of Medical Research, Kaohsiung Medical University Hospital, Kaohsiung, Taiwan; 3 Graduate Institute of Natural Products, College of Pharmacy, Kaohsiung Medical University, Kaohsiung, Taiwan; 4 Research Center for Natural products and Drug Development (CHY), Kaohsiung Medical University, Kaohsiung, Taiwan; 5 Department and Institute of Pharmacology, National Yang-Ming University, Taipei, Taiwan; 6 National Research Institute of Chinese Medicine, Taipei, Taiwan; 7 Department of Life Sciences and Institute of Genome Sciences, College of Life Science, National Yang-Ming University, Taipei, Taiwan; 8 VYM Genome Research Center, National Yang-Ming University, Taipei, Taiwan; 9 Institute of Microbiology and Immunology, National Yang-Ming University, Taipei, Taiwan; 10 Department of Medical Imaging and Radiological Sciences, Kaohsiung Medical University, Kaohsiung, Taiwan; 11 Institute of Medical Science and Technology, National Sun Yat-sen University, Kaohsiung, Taiwan; 12 Graduate Institute of Medicine, College of Medicine, Kaohsiung Medical University, Kaohsiung, Taiwan; 13 Research Center for Environmental Medicine, Kaohsiung Medical University, Kaohsiung, Taiwan; 14 Master Program in Clinical Pharmacogenomics and Pharmacoproteomics, College of Pharmacy, Taipei Medical University, Taipei, Taiwan

## Abstract

Glycine-N-methyl transferase (GNMT) a tumor suppressor for hepatocellular carcinoma (HCC) plays a crucial role in liver homeostasis. Its expression is downregulated in almost all the tumor tissues of HCC while the mechanism of this downregulation is not yet fully understood. Recently, we identified 1,2,3,4,6-penta-O-galloyl-beta-D-glucopyranoside (PGG) as a GNMT promoter enhancer compound in HCC. In this study, we aimed to delineate the mechanism by which PGG enhances GNMT expression and to investigate its effect on GNMT suppression in HCC. Microarray and pathway enrichment analysis revealed that MYC was a major target of PGG. PGG suppressed MYC mRNA and protein expression in Huh7 and Hep G2 cells in a dose- and time-dependent fashion. Furthermore, MYC expression was also reduced in xenograft tumors in PGG treated mice. Moreover, shRNA-mediated knocked-down or pharmacological inhibition of MYC resulted in a significant induction of GNMT promoter activity and endogenous GNMT mRNA expression in Huh7 cells. In contrast, overexpression of MYC significantly inhibited GNMT promoter activity and endogenous GNMT protein expression. In addition, antibodies against MYC effectively precipitated the human GNMT promoter in a chromatin immunoprecipitation assay. Lastly, GNMT expression was negatively correlated with MYC expression in human HCC samples. Interestingly, PGG not only inhibited MYC gene expression but also promoted MYC protein degradation through proteasome-independent pathways. This work reveals a novel anticancer mechanism of PGG via downregulation of MYC expression and establishes a therapeutic rationale for treatment of MYC overexpressing cancers using PGG. Our data also provide a novel mechanistic understanding of GNMT regulation through MYC in the pathogenesis of HCC.

## Introduction

Hepatocellular carcinoma (HCC) remains sixth most prevalent and third most common cause of cancer-related deaths in the globe^1,2^. Despite the new advances in HCC management, the incidence rate is still rising and nearly equals to the mortality rate^3,4^. Therefore, gaining a further understanding of the molecular mechanisms underlying the development of HCC is important to identify novel targets and more effective methods for treatment of HCC.

GNMT a multifunctional protein has a central role in the regulation of one-carbon metabolism in the liver^5,6^. GNMT has protective effects against exposure to various carcinogens including aflatoxins and polycyclic aromatic hydrocarbons^5,7,8^. It has been postulated that GNMT is involved in hepatic detoxification pathways^9^. Recent research has shown that GNMT is involved in cellular signaling cascades that coordinate various cellular processes such as proliferation, differentiation, migration and cell survival by interacting with DEPTOR, NPC2, and PREX2 proteins^10,11^. GNMT is highly expressed in the normal liver and plays a tumor-suppressive function in HCC^5^. The reduced expression of GNMT in human HCC cell lines and tumor tissues of HCC patients was first reported by Chen *et al*. in 1998^12^. Furthermore, other studies demonstrated that GNMT is also downregulated in livers of cirrhotic patients with diverse etiologies, livers from patients with chronic cholestatic liver disease and liver tissues of nonalcoholic fatty liver (NFLD) patients^10,13,14^. In accordance, we described that GNMT expression was downregulated in dietary and genetic mouse models of NFLD^10^. Thus, it is reasonable to propose that GNMT downregulation is an early event in hepatocarcinogenesis. Although the relationship between GNMT downregulation and HCC is well established, the molecular mechanism underlying GNMT downregulation are poorly understood. Only 3 patients with a missense mutation of GNMT gene in the coding region has been reported and shown to affect catalytic activity^15,16^. Importantly, no sequence variations (mutations or deletion) were observed in the coding region of GNMT in sequence analysis suggested that the down-regulation of GNMT may not be caused by gene mutations^17^. In addition, hypermethylation studies showed that 3′ region of the TSS (transcription start site) of GNMT was hypermethylated to some extent in 3 HCC cell lines and seven out of the 35 primary tumors. However, demethylating drug treatment did not show significant induction of GNMT mRNA in HCC cells. Moreover, no significant association between DNA methylation and GNMT mRNA expressions was found in HCC^18^. Collectively, promoter hypermethylation seems not a crucial contributor in transcriptional silencing of GNMT.

Previously, we identified PGG as a GNMT inducer and showed that PGG treatment inhibits HCC cell growth both *in vitro* and *in vivo*^19^. PGG is a natural polyphenolic compound well known for its wide range of biological activities. It’s has been extracted from several medicinal herbs used in traditional treatments of human diseases. PGG possesses potent anticancer activity against numerous cancers and shown to affect various signaling pathways^20,21^. Numerous proteins, including p53, Stat3, Cox-2, VEGFR1, AP-1, SP-1, Nrf-2, NF-κB and MMP-9 have been reported to be involved in the anticancer activity of PGG^22^. However, the precise mechanisms underlying PGG mediated GNMT induction remains elusive. In this study, we investigated the GNMT induction mechanism of PGG to improve the understanding of GNMT downregulation in HCC.

Here we demonstrated that PGG is a potent MYC inhibitor and this inhibition is responsible for GNMT induction by PGG in HCC. Our data also revealed MYC participation in downregulation of GNMT expression in HCC.

## Results

### Global gene expression analysis revealed that MYC is target of PGG

**Figure 1:**
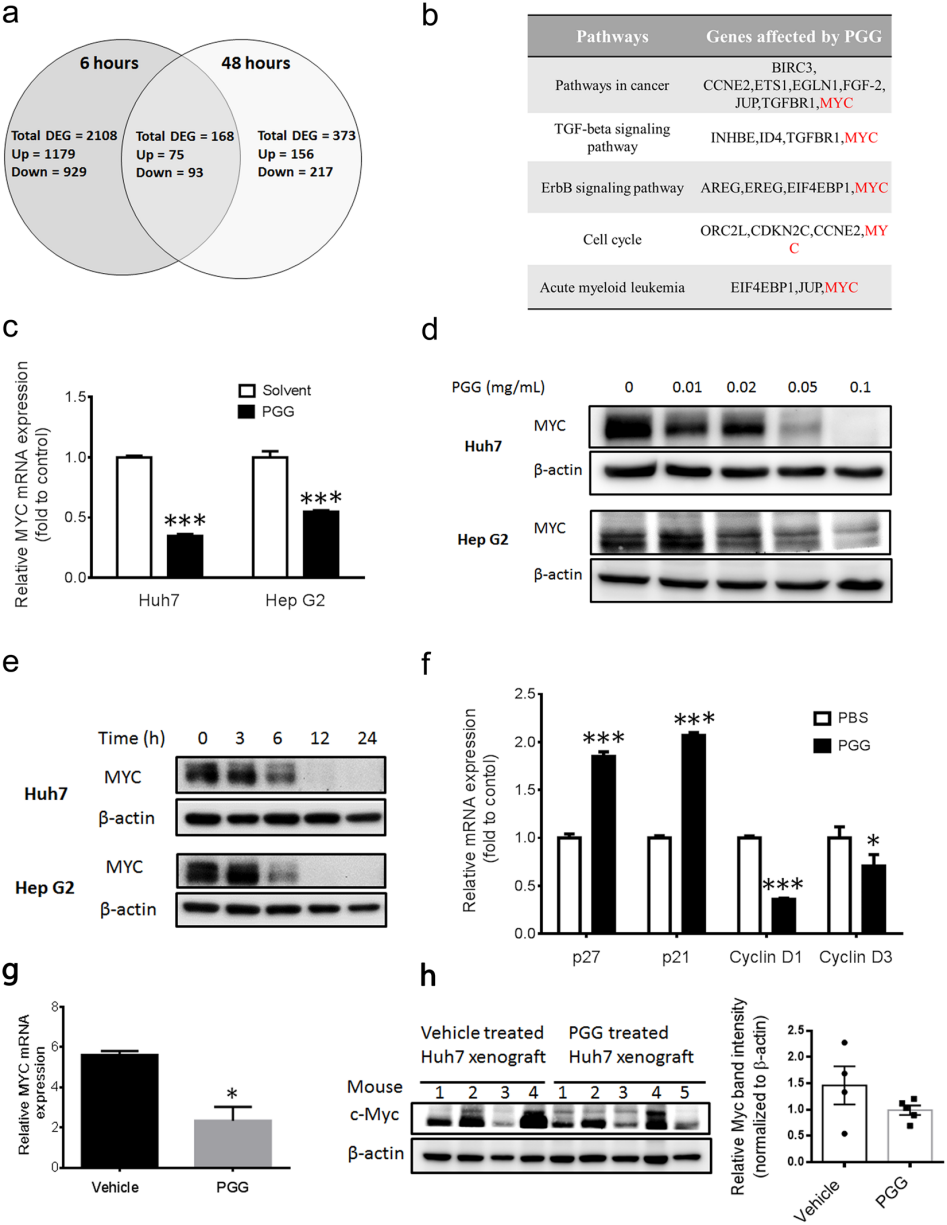
mRNA expression profiling reveals that MYC is target of PGG. (**a**) Genes that were affected by PGG for 1.5-fold (both upregulated and downregulated) were considered as the differentially expressed genes (DEGs). Venn diagram showed that 168 DEGs were persistently affected by PGG from 6 to 48 hours of treatment. **(b)** Pathways and genes identified by DAVID Functional Clustering analysis of the 168 DEGs were shown. Bold red letter indicated MYC is involved in all pathways. **(c)** MYC mRNA expression in PGG (0.1 mg/mL) treated Huh7 and HepG2 cells were determined by qRT-PCR after 24 hours. Data presented as fold to solvent control. The graph shows the means ± SD (n = 3). **(d**,**e)** Immunoblot assay of MYC protein in indicated cells treated with PGG for 24 hours at indicated concentrations (d) and treated with PGG (0.1 mg/mL) for indicated time points (e). β-actin expression was used as loading control. **(f)** Alterations in the mRNA levels of MYC target genes in Huh7 cells 24 hours after PGG (0.1 mg/mL) treatment were detected by qRT-PCR. Data presented as fold to solvent control. The graph shows the means ± SD (n = 3). **(g**,**h)** MYC mRNA (**g**) and protein (**h**) expression in Huh7 xenograft tumor tissues (samples described in previous study^19^) were determined by qRT-PCR and immunoblot assay (n ≥ 4 mice from each group). β-actin expression was used as loading control. Each lane of immunoblot represented the protein sample extracted from Huh7 xenograft tumor of mice in the vehicle-treated group and PGG treated group. Right panel shows quantification of Myc signal intensities in left panel. The graph shows the means ± SEM. ***P < 0.001, *P < 0.05 (Student’s t-test).

The natural compound PGG was previously shown to enhance GNMT promoter activity in HCC^19^. To delineate the mechanism by which PGG enhances GNMT expression, microarray analyses were performed on Huh7 cells treated with either vehicle or PGG. We identified 168 differentially expressed genes that were persistently affected by PGG from 6 hours to 48 hours and were subjected to pathway enrichment analysis (Table 1a and Supplementary Table S1). Five pathways including, pathways in cancer, TGF-beta signaling pathway, ErbB signaling pathway, cell cycle and acute myeloid leukemia pathways were identified. It is important to note that MYC was the only gene that involved in all pathways (Fig. 1b). The effect of PGG on MYC expression was verified by qRT-PCR (Fig. 1c). Furthermore, PGG inhibited MYC expression in Huh7 and Hep G2 cells in a dose and time-dependent fashion (Fig. 1d,e). Moreover, the functional inhibition of MYC by PGG was evidenced by qRT-PCR analyses of MYC target gene such as p21, p27, cyclin D1 and D3 (Fig. 1f). Consistent with *in vitro* results, MYC mRNA and protein expression were remarkably reduced in Huh7 xenograft tumors in PGG treated mice (Fig. 1g,h). These results demonstrated that PGG suppresses MYC expression in HCC cells.

### MYC inhibits GNMT expression in HCC

**Figure 2:**
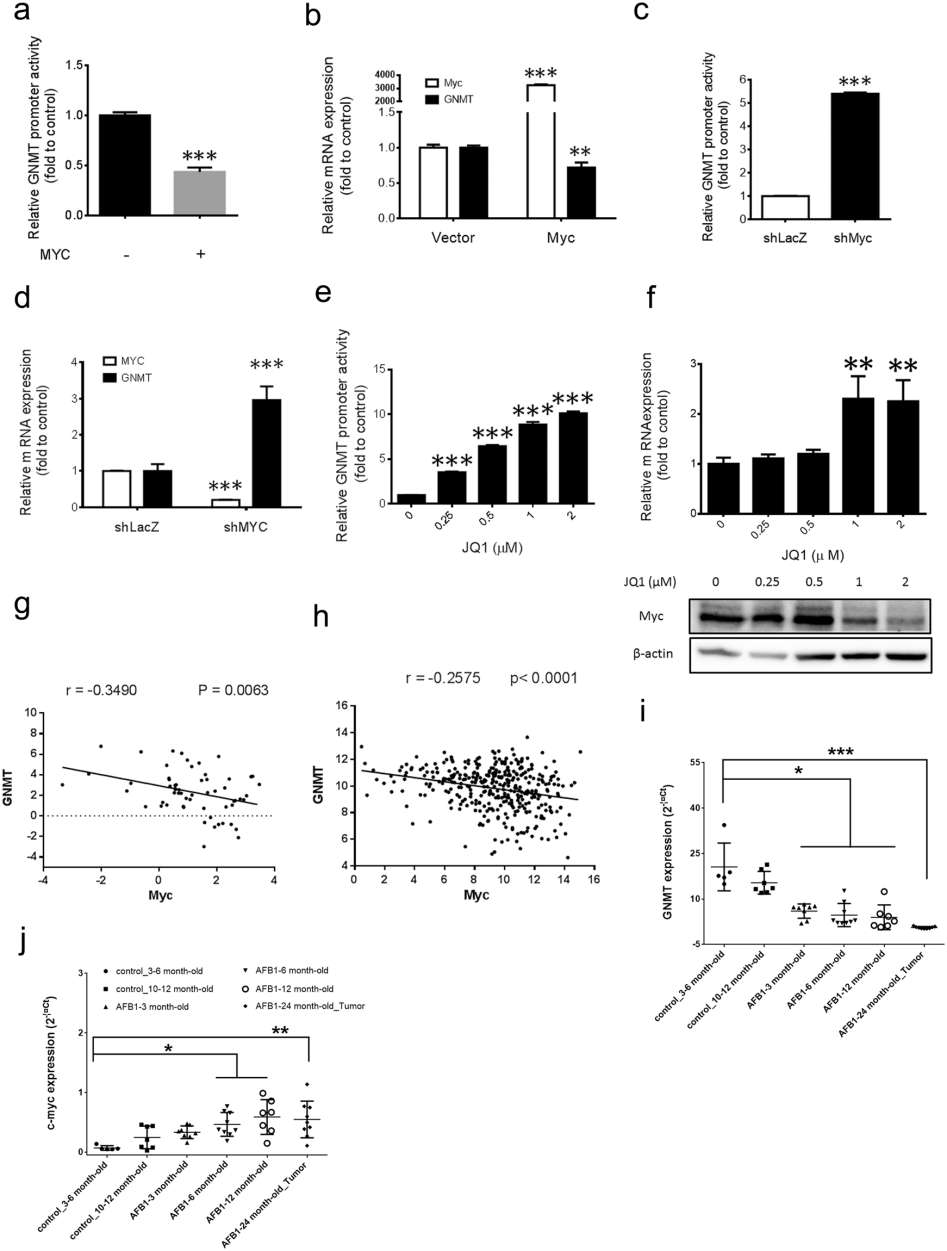
MYC inhibits GNMT expression. **(a)** Huh7 cells were co-transfected with the GNMT promoter reporter, TK renilla reporter and pcDNA-MYC or control vector plasmid for 72 hours, then harvested for luciferase assay. Luciferase activity were measured and normalized to renilla reporter activity. Data were represented as fold to vector control. The graph shows the means ± SD (n = 3). **(b)** Huh7 cells were transfected with the pcDNA-MYC or control vector plasmid. Cells were harvested after 72 hours of transfection and relative level of MYC and GNMT mRNA were determined by qRT-PCR. Data normalized to internal control and presented as fold to vector control. The graph shows the means ± SD (n = 3). **(c)** Huh7 cells were co-transfected with the GNMT promoter reporter, TK renilla reporter and shMYC or shLacZ plasmids for 72 hours, then harvested for luciferase assay. Data were represented as in (**a**). The graph shows the means ± SD (n = 3). **(d)** Relative GNMT and MYC mRNA level in Huh7-shMYC and Huh7-shLacZ stable cells were determined by qRT-PCR. Data were represented as fold to Huh7-shLacZ control group. The graph shows the means ± SD (n = 3). **(e)** Effect of indicated concentrations of JQ1 on GNMT promoter expression in H7GPL cells after 48 hours of treatment. Relative luciferase activity Relative luciferase activity was calculated by normalizing luciferase activity to cell viability and presented as fold to control. Results are means ± SD (n = 3). **(f)** Effect of indicated concentrations of JQ1 on GNMT mRNA (upper panel) expression in Huh7 cells after 48 hours of treatment. Lower panel shows the MYC protein expression after JQ1 treatment. Results are means ± SD (n = 3). **(g)** The mRNA levels of MYC and GNMT in the human HCC tumor samples were determined by qRT-PCR (n = 60). **(h)** TCGA data set (n = 373) were assessed for MYC and GNMT correlation using the Pearson’s correlation analysis. **(i,j)** The mRNA expression of GNMT (**i**) and Myc (**j**) in the liver tissues from mice challenged with AFB1 were determined by qRT-PCR. Results are means ± SD. ***P < 0.001, **P < 0.01; *P < 0.05 (Student’s t-test).

MYC is a transcription factor known for both activation and repression of the genes^23^. Therefore, we hypothesized that PGG might induce GNMT promoter activity by MYC suppression. To test this hypothesis, we examined the effect of MYC in the regulation of GNMT expression using over-expression and silencing studies. Overexpression of MYC significantly inhibited GNMT promoter activity and endogenous GNMT mRNA and protein expression (Fig. 2a,b and Supplementary Fig. S1a,b). These results were consistent with the finding that MYC inhibition by shRNA mediated knock down or by chemical inhibitor-JQ-1 significantly enhanced GNMT promoter activity and its mRNA level (Fig. 2c–f). Based on *in vitro* results, we further examined whether MYC mRNA expression is associated with GNMT in tumors from HCC patients (n = 60). qRT-PCR result analysis showed that the expression levels of MYC mRNA in HCC tissues were significantly negatively correlated with GNMT mRNA levels as determined by Pearson’s correlation (Fig. 2g). Furthermore, a similar negative correlation between MYC and GNMT obtained in the TCGA liver cancer dataset (n = 371) (Fig. 2h) (http://r2.amc.nl). Finally, using the aflatoxin B1 (AFB1) -induced mouse model of HCC we confirmed that MYC mRNA expression is associated with GNMT *in vivo*. As shown in (Fig. 2i,j) there was a significant increase in MYC mRNA expression with a significant decrease in GNMT mRNA expression in AFB1 induced HCC development in mice. Taken together these findings suggested that MYC negatively regulates GNMT expression in HCC.

### MYC protein interacts with the promoter of GNMT

**Figure 3:**
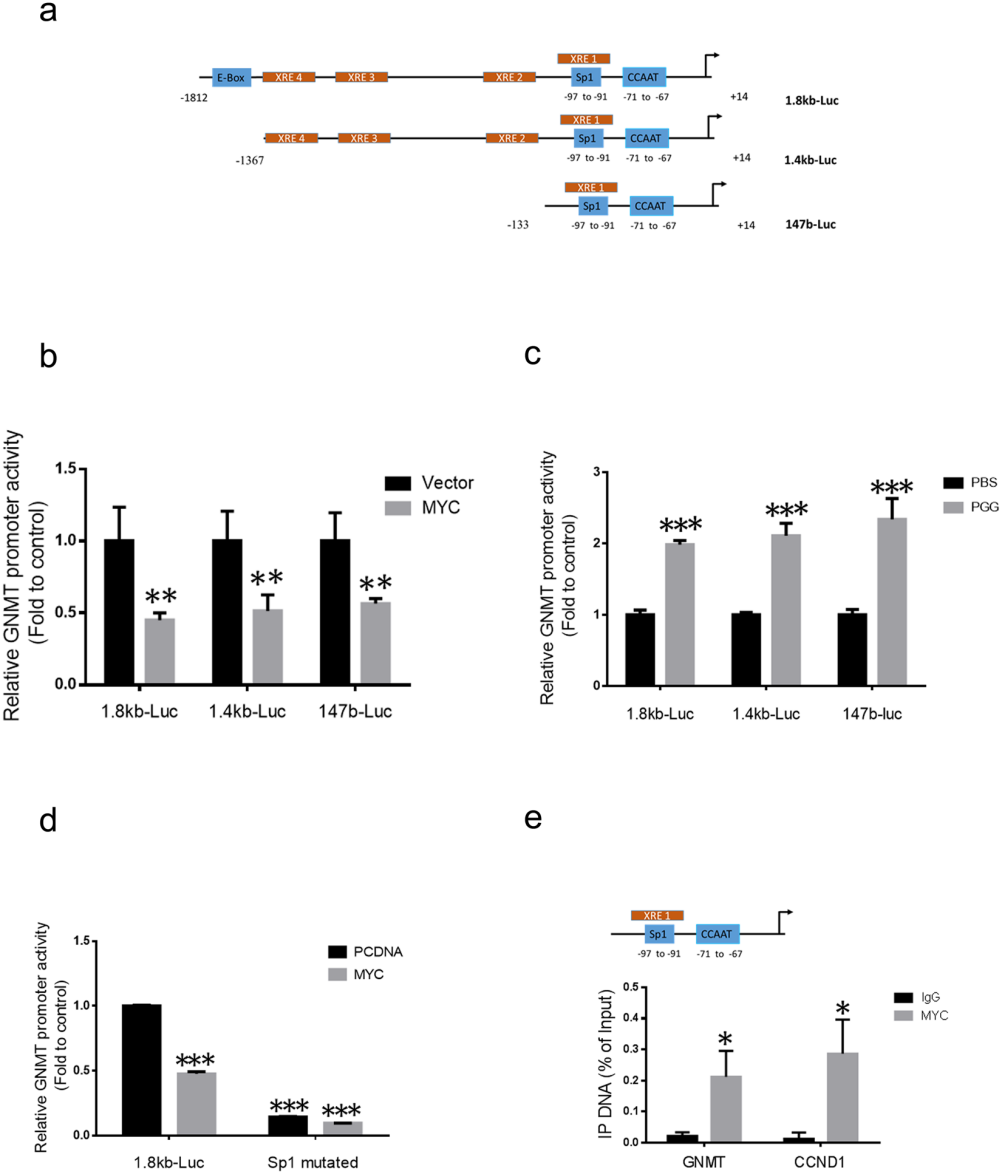
MYC interacts with the promoter of GNMT. **(a)** Schematic representation of the GNMT promoter-driven luciferase reporter constructs (1.8Kb-Luc; −1812/+14) and two 5′-deletion mutant promoter constructs (1.4Kb-Luc; −1367/+14, deleted of E-box and 147b-Luc; −133/+14, the core promoter). The indicated constructs are described previously^26^. **(b)** Huh7 cells transiently co-transfected with the reporter plasmids containing the 5′-flanking region of human GNMT promoter described in (**a**) with TK renilla reporter and pcDNA-MYC or control vector plasmids. Luciferase activity was measured 72 hours post-transfection and normalized to renilla reporter activity. Data were represented as fold to vector control. The graph shows the means ± SD (n = 3). **(c)** Huh7 cells transiently co-transfected with the reporter plasmids containing the 5′-flanking region of human GNMT promoter described in (**a**) with TK renilla reporter for 72 hours and treated with PGG or PBS solvent control. Luciferase activity was measured 24 hours after treatment and normalized to renilla reporter activity. Data were represented as fold to solvent control. The graph shows the means ± SD (n = 3). **(d)** Huh7 cells were co-transfected with the GNMT promoter reporter, SP1 mutated GNMT promoter reporter (described previously), TK renilla reporter and pcDNA-MYC or control vector plasmid for 72 hours, then harvested for luciferase assay. Data were measured and represented as described above. The graph shows the means ± SD (n = 3). **(e)** ChIP-qPCR analysis was performed in Huh7 cells. Enrichment of the GNMT promoter region (containing the core promoter −133/+14) and CCND1 promoter region (containing MYC response element) were calculated by qPCR quantification normalized to input. Results are means ± SD (n = 3). **P < 0.01; *P < 0.05 (Student’s t-test).

Next, we examined whether MYC would repress transcription of the GNMT. The PROMO (TRANSFAC v8.3) program was used to predict MYC binding sites in the 1.8Kb GNMT promoter fragment (−1812/+14)^24^. Only one putative MYC binding site (E-box) was identified at distal region (−1775/−1780). In addition, MYC could also act as transcriptional repressor via binding to transcriptional initiator (Inr) elements or SP1 transcription factor in core promoters, which can both be found in GNMT core promoter; SP1 binding site: (−97/−91); CCAAT box: (−71/−67)^25,26^. Hence, to determine whether the distal E-box or the core promoter plays a crucial role in MYC-mediated GNMT suppression, three GNMT promoter-luciferase reporters-the 1.8Kb-Luc, 1.4Kb-Luc (−1367/+14, deleted of E-box) and 147b-Luc (−133/+14, the core promoter) were used (Fig. 3a). Overexpression of MYC was able to repress all GNMT promoter reporters with the same magnitude (Fig. 3b). Furthermore, as expected PGG treatment able to induce all GNMT promoter reporters with the same magnitude in Huh7 cells expressing endogenous MYC (Fig. 3c). These results suggested that endogenous MYC level act on the promoter, PGG treatment inhibit Myc and reverse repressive effect of Myc on GNMT promoter. Moreover, we also mutated core putative SP1 binding cite in GNMT promoter and examined the effect of Myc overexpression on SP1 mutant construct^26^. Mutation of the SP1 site resulted in a significant decrease of luciferase activity compared with the non-mutated promoter and interferes with the suppressive effect of Myc overexpression on GNMT promoter expression (Fig. 3d). These results demonstrated that the suppressive effect of MYC on GNMT transcription relied on the core promoter rather than the distal E-box. To further endorse the transcriptional regulation of GNMT gene by MYC, we carried chromatin immunoprecipitation assays in Huh7 cells. A significant enrichment of MYC chromatin immunoprecipitate (MYC IP) compared with the IgG control was observed using ChIP-PCR primers designed against the GNMT core promoter (−133/+14). Primers against the promoter of CCND1 gene known to be the target of MYC was used as positive control and yielded comparable enrichment (Fig. 3e). These data indicated that MYC protein interacts with the GNMT core promoter and GNMT is a target gene of MYC.

### PGG affects GNMT promoter activity through inhibition of MYC expression

**Figure 4:**
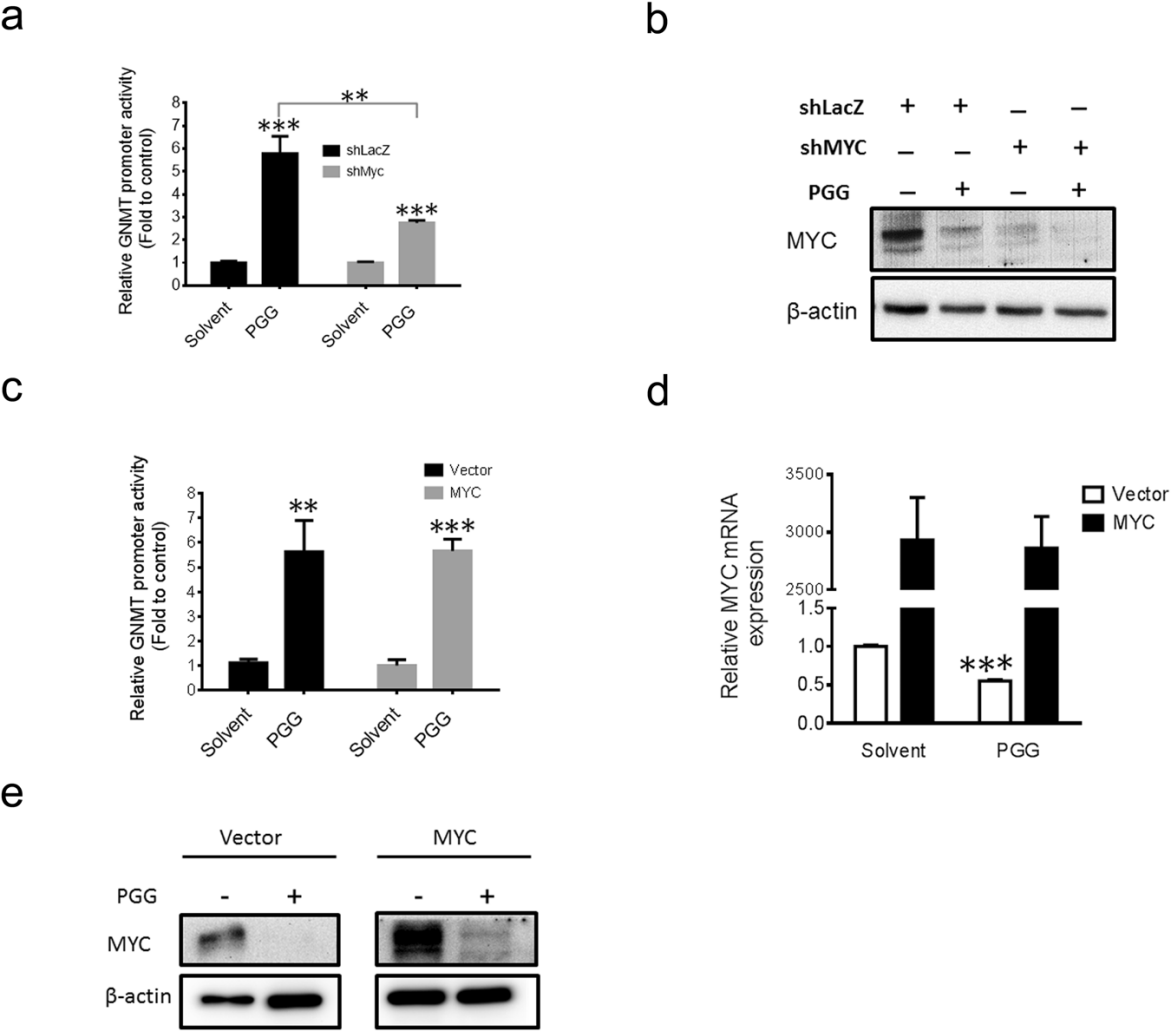
PGG affects GNMT promoter activity through inhibition of MYC expression. **(a)** Huh7 cells were co-transfected with the GNMT promoter reporter, TK renilla reporter and shMYC or shLacZ plasmids for 72 hours. Cells were treated with PGG (0.1 mg/mL) or solvent for 24 hours and harvested for luciferase assay and immunoblot analysis. Luciferase activity were measured and normalized to renilla reporter activity. Data were represented as fold to solvent control. The graph shows the means ± SD (n = 3). (**b**) Protein level of MYC in above mentioned cells were measured by Immunoblot assay. β-actin expression was used as loading control. **(c)** Huh7 cells were co-transfected with the GNMT promoter reporter, TK renilla reporter and pcDNA-MYC or control vector plasmid for 72 hours. Cells were treated with PGG (0.1 mg/mL) or solvent for 24 hours and harvested for luciferase assay. Luciferase activity were measured and normalized to renilla reporter activity. Data were represented as fold to solvent control. The graph shows the means ± SD (n = 3) **(d)** MYC mRNA expression in above mentioned cells were determined by qRT-PCR. Data normalized to internal control and presented as fold to vector control. The graph shows the means ± SD (n = 3). **(e)** Protein level of MYC in above mentioned cells were measured by Immunoblot assay. β-actin expression was used as loading control. ***P < 0.001, **P < 0.05 (Student’s t-test).

To confirm that the GNMT promoter induced by the PGG was indeed caused by suppression of MYC expression, we studied whether knocking down of MYC would impair the induction of GNMT promoter activity by PGG treatment. As shown in Fig. 4a,b, knocked down of MYC significantly reduce the induction of GNMT promoter activity by PGG. These data indicated that MYC downregulation has a major role in PGG induced GNMT promoter expression in Huh7 cells. Next, we asked whether ectopic expression of MYC would rescue the induction of GNMT promoter by PGG. Surprisingly, our results showed that overexpression of MYC did not revert PGG-induced GNMT promoter activity (Fig. 4c). Interestingly, ectopic expression of MYC was able to rescue PGG-induced MYC mRNA suppression but was not able to prevent PGG-induced MYC protein depletion in Huh7 cells (Fig. 4d,e). These results suggested that PGG not only effects MYC at mRNA level but also decreases MYC protein stability.

### PGG induces proteasome-independent degradation of MYC

**Figure 5:**
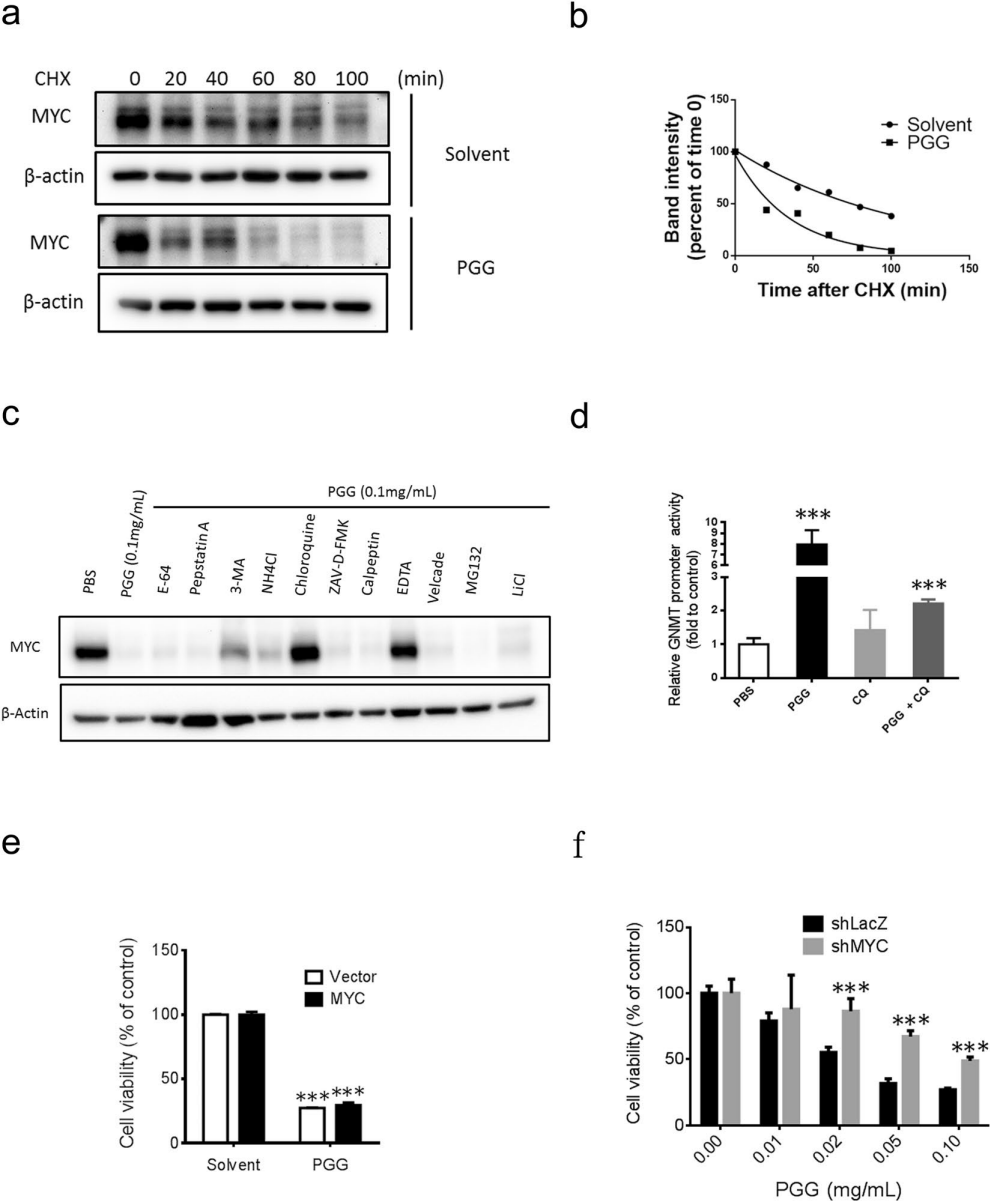
PGG induced decreases of MYC through proteasome independent degradation in Huh7 cells. **(a)** Huh7 cells were treated with PGG (0.1 mg/mL) for 3 hours and then co-exposed to cycloheximide (CHX 50 ug/ml) for indicated time intervals and harvested for immunoblot analysis for MYC expression. β-actin expression was used as loading control. **(b)** Graph shows quantification of MYC signal intensities in (**a**). **(c)** MYC overexpressed Huh7 cells were pre-incubated with indicated compounds for 1 hour and then co-treated with PGG for 24 hours. MYC protein level was determined by immunoblotting. β-actin expression was used as loading control. **(d)** Effect of indicated compounds (PGG 0.1 mg/mL, chloroquine (CQ) 100 μM) on GNMT promoter expression in H7GPL cells after 24 hours of treatment. Relative luciferase activity was calculated by normalizing luciferase activity to cell viability and presented as fold to control. Results are means ± SD (n = 3). **(e)** MYC overexpressed and control-Huh7 cells were treated with PGG (0.1 mg/mL) or solvent for 72 hours. Cell viability was determined by alamarBlue® viability assay. Results are means ± SD (n = 3). **(f)** Huh7-shLacZ and Huh7-shMYC stable cells were treated with various concentrations of PGG for 72 hours. Cell viability were determined by using MultiTox-Glo Multiplex Cytotoxicity Assay kit. The percentages of viable cells compared with the cells without PGG treatment are plotted. Results are means ± SD (n = 3). ***P < 0.001 (Student’s t-test).

Because PGG modulates MYC protein stability, therefore, cycloheximide chase assay was performed to determine the degradation kinetics of MYC upon PGG treatment. In the presence of PGG, the half-life of MYC protein reduced remarkably (Fig. 5a,b). These results indicated that downregulation of MYC protein by PGG occurred through enhanced protein degradation. Next, we determined the proteolytic pathway involved in PGG-induced MYC degradation. Previous reports have shown that the proteolysis of MYC is mediated through ubiquitin-proteasome pathways^27^. Therefore, we examined whether PGG induced destabilization of MYC results from increased proteasomal degradation. Immunoblot results showed that proteasome inhibitor (MG132) and velcade did not prevent PGG-induced MYC protein depletion in Huh7 cells (Supplementary Fig. S2). Because PGG inhibits MYC at mRNA and protein levels accordingly we used MYC overexpression system for further study to rule out inhibition at the transcriptional level and evaluated the effects of various protein degradation inhibitors on PGG-mediated MYC depletion. Proteasome inhibitors (MG132 and velcade), GSK 3β inhibitor (LiCl), protease inhibitors (E-64 and pepstatinA), calpain protease inhibitor (calpeptin), apoptosis inhibitor (ZAV-D-FMK) and lysosomes inhibitors (NH4Cl and 3-MA) did not prevent the PGG-induced MYC depletion, while lysosomal inhibitor chloroquine and ion chelator EDTA completely rescued PGG mediated MYC depletion in MYC overexpressed cells (Fig. 5c). Accordingly, our findings indicated that PGG induces MYC degradation through proteasome-independent mechanisms in Huh7 cells. Moreover, co-treatment of chloroquine with PGG revert PGG-induced GNMT promoter activity in H7GPL cells (Fig. 5d). Finally, we examined the role of MYC inhibition in the anti-HCC activity of PGG. Because PGG interferes with the protein stability of MYC, therefore, ectopic expression of MYC did not protect Huh7 cells against PGG-induced cytotoxicity (Fig. 5e). Next, we used a knockdown system to reveal the role of MYC in the PGG-induced cytotoxicity. The significant rescue effect on the cytotoxicity induced by PGG in MYC knockdown cells (Fig. 5f) indicated that PGG affects Huh7 cell growth primarily through MYC downregulation. In addition, we determined whether PGG-induced inhibition of MYC is a universal phenomenon in tumor cells. Results from immunoblot analysis showed that PGG treatment also reduced MYC expression in other human cancer cell lines, including PC-3 (prostate cancer) and HL-60 (leukemia) (Supplementary Fig. S3).

## Discussion

**Figure 6:**
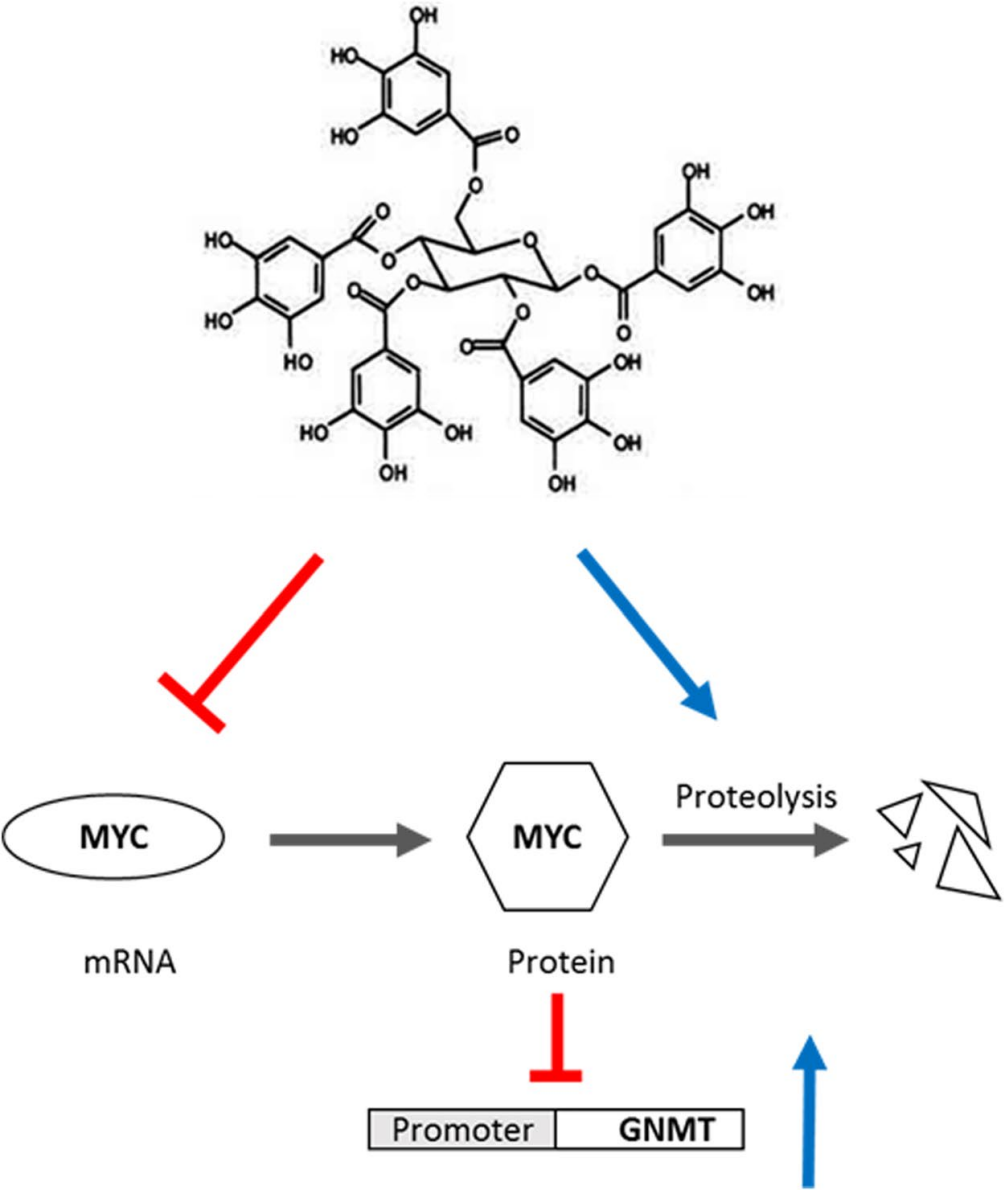
The proposed molecular mechanisms of GNMT induction of PGG. The model shows that PGG inhibiting MYC mRNA and inducing MYC proteolysis and therefore inducing GNMT expression through MYC inhibition. The red arrows denote suppression whereas the blue arrow denotes activation.

Inactivation of a tumor suppressor is an important step in the complex process of hepatocarcinogenesis^28^. GNMT exhibits tumor-suppressive role and its expression highly downregulated in human HCC^5^. Most studies have focused on the functional role of GNMT downregulation in liver disease that leads to HCC. Few studies have addressed the regulation of GNMT gene expression and the mechanism of GNMT downregulation in HCC. It has been reported that nuclear factor-Y (NF-Y) and liver homolog receptor 1 (LHR-1) transcription factors binds to GNMT promoter and activate GNMT^26,29^. However, the contribution of these factors in GNMT downregulation is not studied in HCC. Huidobro *et al*. suggested that DNA hypermethylation is partly responsible for the transcriptional repression of GNMT in HCC. In contrast, demethylating agent (AdC) did not induce GNMT expression in HCC cells significantly (less than 1.5-fold induction)^18^. In our previous study, we identified PGG as a GNMT inducer using GNMT promoter based assay^19^. In this study, we attempted to explore the mechanism of action by which PGG induces GNMT expression in HCC. We found that MYC was a molecular target of PGG. Furthermore, we showed that MYC transcriptionally regulates GNMT by interacting with the core promoter of GNMT. A negative correlation between GNMT and MYC expression was also observed in human HCC specimen and TCGA liver cancer dataset. Moreover, we demonstrated that PGG enhances GNMT promoter activity via inhibition of MYC expression in HCC (Fig. 6a). To the best of our knowledge, this is the first report showing that MYC is involved in GNMT suppression in HCC. Retinoic acid and dexamethasone have been reported to up-regulate GNMT in rat by an unknown mechanism^30,31^. Interestingly, previous studies demonstrated that retinoic acid reduced MYC in rat liver as well as in fetal rat hepatocytes^32,33^. Furthermore, retinoic acid and dexamethasone inhibit MYC expression in various cancers^34^. These reports coincided with our findings in this study. We speculate that retinoic acid and dexamethasone could promote GNTM expression in part through MYC suppression.

The transcriptional activation function of MYC mainly relies on its binding to the E-box motif (CACGTG), while MYC acts as a transcriptional repressor through two mechanisms: by binding to the transcriptional initiator (Inr) element in the promoter; or by binding and inhibiting Sp1 transcriptional activity^23,25,35^. We identified an E-box element at distal region; and one Sp1 site and one Inr element in the core promoter region of the 1.8Kb GNMT 5′ flanking fragment. Using GNMT promoter–luciferase deletion and SP1 mutated constructs we showed that the distal E-box is not associated with MYC-mediated GNMT downregulation. This also indicates that the interaction between MYC and Sp1 or Inr element may contribute to GNMT promoter downregulation in HCC. Further investigation is needed to address the Inr dependent and independent inhibition of GNMT promoter by the MYC. In addition, our recent report showed that histone deacetylase inhibitors TSA (Trichostatin A) induced GNMT promoter activity and mRNA expression in Huh7 cells^19^. Interestingly, the combination of PGG with TSA resulted in higher induction of GNMT mRNA expression compared to any of single treatments alone in Huh7 cells (Supplementary Fig. S4). These data suggested that MYC-mediated repression and repression via histone deacetylation are two independent, important mechanisms of GNMT suppression in HCC.

PGG exhibits a potent antitumor effect against a variety of cancers, including breast cancer, sarcoma, leukemia, melanoma, prostate cancer, breast cancer, and lung cancer *in vitro* and *in vivo* models without host toxicity. PGG has been described to exert its anticancer activity by antioxidation, anti-inflammation, anti-angiogenesis, inhibiting DNA replicative synthesis, arresting cells in G1 and S phase, autophagy-mediated senescence, and inducing apoptosis^22^. In the present study, we found that PGG is a novel MYC inhibitor that inhibits MYC gene expression and enhances the degradation of the MYC protein. It is important to note that MYC is a pivotal regulator of many cellular processes such as cell cycle control, metabolic homeostasis and autophagy, and cell survival^36^. Thus, PGG induced G1 cell cycle arrest and apoptosis in cancer cells can also be explained by MYC inhibition. Therefore, it is reasonable to propose that the suppression of MYC expression, as well as its downstream targets by PGG, plays an important role in its anticancer activity. Moreover, using SYBR green real-time RT–PCR we verified the effect of PGG on the expression of genes (PLK1, CDK6, p57, p18) identified in microarray involved in cell cycle and apoptosis (Supplementary Fig. S5). Our findings provided a new insight into the mechanism of action of PGG in cancer treatment. Additionally, the inhibitory effect of PGG on MYC expression can also be observed in prostate cancer and leukemia cell lines (Supplementary Fig. S3). Therefore, these results bring a rationale for the prospective use of PGG in the treatment of cancers with MYC amplification/overexpression.

The MYC is a transcription factor crucial for normal cell functions including cell growth, proliferation, metabolism, protein biosynthesis, and microRNA regulations^36^. Overexpression of MYC contributes to development as well as the progression of almost all the cancer types including HCC^37^. Most cases of HCC overexpresses MYC and its upregulation in hepatic cells leads to the development of HCC^38^. Due to its oncogenic role, MYC is an attractive molecular target for tumor intervention therapy^39^. Researchers tried to identify MYC inhibitors in recent years^40^. So far, two classes of MYC inhibitors (direct and indirect) have been identified. Direct MYC inhibitors, such as 10058-F4, disrupt Myc-Max dimerization. Direct MYC inhibition has proven to be a challenging task due to lack of obvious binding pockets for small molecules and more importantly, the direct targeting compound has shown very little success for clinical use^41^. Indirect inhibitors, such as JQ1, inhibit MYC transcription without affecting Myc-Max heterodimerization. However, recent studies have shown that various cancers have acquired resistance to JQ1 treatment^42^. The other indirect inhibitors, such as dihydroartemisinin, work by modulating the MYC protein stability^43^. It is interesting to note that PGG suppresses MYC expression at both transcriptional and post-translational level. Ubiquitin-dependent proteasome pathways using several E3 ligases is the most frequent pathway responsible for MYC protein degradation^27^. Li *et al*. showed that proteasome activator REGγ also regulates protein stability of MYC by ubiquitin-independent pathways^44^. In addition, calcium-activated calpain-dependent cytoplasmic cleavage of MYC has been reported to be responsible for MYC degradation as well^45^. Thus it is important to know that PGG accelerated MYC protein degradation through a proteasome- as well as calpain-independent mechanisms. Interestingly, pretreatment with chloroquine and EDTA showed significant rescue in PGG-mediated MYC repression in MYC overexpressed cells providing a direction for further studies. Chloroquine is known as a lysosomal inhibitor, however, the addition of other lysosomal inhibitors (3-MA and NH4Cl) do not show significant rescue in PGG mediated MYC repression, suggesting that the other unknown mechanism of chloroquine might be responsible for this rescue^46^. The possible mechanisms underlying MYC inhibition by PGG is under investigation.

It is interesting to note that overexpression of GNMT extends the lifespan in Drosophila^47^. Interestingly, Hofmann *et al*. demonstrated that reduction of MYC expression in MYC haploinsufficient (Myc+/−) mice promotes longevity^48^. Even more interesting is that PGG has been shown to extend the life span of *Caenorhabditis elegans*^49^. Together with our finding, it is reasonable to hypothesize that induction of GNMT and alteration of the SAM (S-adenosdyl-methionine) metabolism contributes to the longevity of the Myc+/− mice. In addition, it is also possible that PGG increases lifespan through MYC down-regulation and subsequent alteration of the SAM metabolism. Thus, further studies of PGG and its suppressive effect on MYC is warranted and maybe beneficial to human medicine.

In summary, this study demonstrated the usefulness of gene enhancer or repressor compounds as a tool for identifying the gene regulation mechanisms in cancer. Interestingly, the next questions that arise here are how does PGG induce MYC protein degradation? Is MYC stability is regulated by proteasome-independent pathways or is there a novel MYC protein degradation mechanism? Do these mechanisms exist in normal cells or only in cancer cells? Answers to these questions will contribute to better understanding of cancer biology and drug discovery.

## Methods

### Cell culture and reagents

Huh7 cells and Hep G2 cells were cultured in complete Dulbecco’s modified Eagle’s medium (DMEM) (Gibco BRL, Grand Island, NY). DMEM supplemented with 10% heat-inactivated fetal bovine serum (HyClone, Logan, UT, USA), penicillin (100 U/mL), streptomycin (100 μg/mL), nonessential amino acids (0.1 mM/L), and L-glutamine (2 mM/L) (Thermo Fisher Scientific, Waltham, MA) at 37 °C in 5% CO2. Stable cell established by lentiviral-system including H7GPL^19^, Huh7-shLacZ, Huh7-shMYC were grown in DMEM supplemented with 1 μg/mL puromycin. The cyclohexmide, 3-methyladenine, MG132, chloroquine diphosphate, E-64, cycloheximide (CHX) pepstatin A and (+/−) JQ-1 were purchased from Sigma-Aldrich (St Louis, MO, USA), Z-AVD-FMK from Selleckchem.com, calpeptin from Millipore (Billerica, MA, USA) and PGG obtained from The One Biopharmaceutical Co., Ltd. (Hsinchu County, Taiwan) as described previously^19^.

### Plasmids and transfections

The pGL3-luciferase constructs containing the GNMT promoter region with the deletions (1.8Kb-Luc, 1.4Kb-Luc (−1367/+14, deleted of E-box) and 147b-Luc (−133/+14, the core promoter) were constructed and described previously^26^. Plasmids for lentivirus production (pCMV-ΔR8.91 and pMD.G), shRNAs for MYC (MYC-lentiviral shRNA; 5′ CCTGAGACAGATCAGCAACAA 3′) and control plasmid for the RNA interference (pLKO.1-shLacZ) were obtained from the National RNAi Core Facility (Academia Sinica, Taipei, Taiwan). The pcDNA-MYC plasmid and vector control plasmid for overexpression experiments were provided by Dr Shih-Ping Liu (China Medical University, Taichung, Taiwan). The Renilla luciferase control reporter vector [(Promega, Madison, WI, USA)], was used for normalizing transfection efficiency. Plasmid DNAs were transfected by using TurboFect Reagent (Fermentas, Hanover, MD) and Lipofectamine 3000 (Life Technologies, Mulgrave, Australia) according to the manufacturer recommendations. Lentivirus production, transduction and generation of stable cell lines were performed as described previously^50^.

### Microarray analysis

Huh7 cells were treated with PGG (0.1 mg/mL) for 6 hours, 48 hours and solvent 48 hours in triplicates. Total RNA were extracted as described above and were used for microarray gene expression analysis (National Microarray & Gene Expression Analysis Core Facility of the National Research Program for Genomic Medicine at Yang-Ming University, Taipei, Taiwan). Array hybridization were performed according to the Affymetrix Gene chip expression analysis technical manual. Raw data was normalized by Exprssion ConsoleTM (Affymetrix inc., USA). Significant changed probe sets between treatment and control were detected by t-test statistic (q-value < 0.005 and Mean-diff > 1.5). Differentially expressed genes presented by selected probe sets were annotated and pathway enrichment analysis was performed by using DAVID Bioinformatics online tools (Database for Annotation, Visualization and Integrated Discovery; http://david.abcc.ncifcrf.gov/)^48^. The microarray data have been submitted to the Gene Expression Omnibus (GEO) public database at NCBI (Accession No. GSE75024). http://www.ncbi.nlm.nih.gov/geo/query/acc.cgi?token=axkzwoyojlqbhel&acc=GSE75024.

### Chromatin immunoprecipitation

ChIP was performed according to the protocol described before^51^. Concisely, chromatin DNA from formaldehyde-fixed (1% v/v) Huh7 cells was harvested and immunoprecipitated. Immunoprecipitations was performed using ChIP grade anti-MYC (Abcam, UK) antibody and rabbit non-immune serum IgG (Alpha Diagnostic International, San Antonio, Texas, USA). The immunoprecipitate was PCR amplified using promoter-specific primers. Sequences for primers are listed in Supplementary Table S2. As positive controls primers directed against the established MYC target genes CCND1 was used in the ChIP assay.

### Quantitative real-time PCR (qRT-PCR)

Total RNA was isolated by using Tri Reagent (Sigma-Aldrich) and cDNA was synthesized using a Super Script II Reverse Transcriptase Kit (Invitrogen Inc., Carlsbad, CA, USA). PCR was performed on an ABI StepOne Plus System (Applied Biosystems, Foster City, CA) with the LightCycler® First Start DNA Master SYBR Green I reagent (Roche Diagnostics, Basel, Switzerland). The mRNA level was normalized using the TBP as an internal control to calculate relative expression. The primers used in this study shown in Supplementary Table S2.

### Immunoblotting

Cells or xenograft tumors were lysed by RIPA lysis buffer (50 mM Tris [pH 7.5], 150 mM NaCl, 1% Triton X-100, 0.1% SDS, 0.5% sodium deoxycholate, supplemented with protease inhibitor cocktail (Roche, Mannheim, Germany) and phosphatase inhibitors (1 mM NaF, 5 mM NaPPi, and 10 mM Na3VO4). Immunoblotting was carried out as described previously^50^. The anti-GNMT (14-1, YMAC Bio Tech, Taiwan), anti-MYC (D84C12, cell signaling and 46–0603, Invitrogen) and anti-β-actin (AC-15, sigma-aldrich) antibodies were used in this study.

### Luciferase Reporter Assays and cell viability assay

The detailed methods of luciferase assay have been described previously^19^. Briefly, cells were lysed in Passive Lysis Buffer (Promega) and luciferase activity was assayed using the Luciferase Assay System (Promega) and Dual-Luciferase Reporter Assay System (Promega) according to the manufacturer’s recommendations. AlamarBlue® assay (AbD serotec, Raleigh, NC, USA) and MultiTox-Fluor multiplex cytotoxicity assay (Promega) were used to evaluate the cytotoxicity according to the manufacturer’s instructions.

### Ethical Approval for Hepatocellular carcinoma specimens

For qRT-PCR, Sixty liver cancer tissue samples were obtained from the Taiwan Liver Cancer Network (TLCN, http://tlcn.nhri.org.tw/TLCN/index.jsp). The liver tumor tissues removed from the patients and there is no chemotherapy treatment before surgery; thus, the pathology stage can represent the status of tumor progression. Human HCC specimen study was approved by the Institutional Review Board of Kaohsiung Medical University and the user committee of TLCN. The detailed information was shown in Table 1.

**Table 1:** Main clinical and histopathologic features of 60 HCC patients.

N = 60	N (%)
Age	61.5 (33–86)
Gender	
Male	50 (83.3)
Female	10 (16.7)
Viral infection*	
HBV	29 (48.3)
HCV	30 (50.0)
Both HBV and HCV	1 (1.7)
Cirrhosis	
Negative	33 (55.0)
Positive	27 (45.0)
TNM Stage	
I	26 (43.3)
II	24 (40.0)
IIIA	5 (8.3)
IIIB	4 (6.7)
IIIC	1 (1.7)

^*^HBV, patients with HBV sAg (+); HCV, patients with anti HCV antibody (+).

### Huh7 xenograft tumor and aflatoxin B1-induced HCC mouse models

The xenograft assay was performed and described previously^19^. In brief, The Huh7 cells were injected subcutaneously in the right flank of Balb/c nude mice (2*10^6^). Huh7 tumor-bearing mice with the size of approximately 100 mm^3^ were divided into two groups, with five mice per group and treated with PGG 300 mg/kg body weight (mpk) and drug vehicle. The tumor growth was monitored by using a Vernier caliper throughout the experiment. Tumors were lysed and used for immunoblot analysis as described above in immunoblot section. Data are presented as the mean of tumor volume ± SEM.

For aflatoxin B1 (AFB1)-induced HCC mouse model, wild-type male mice (129/Sv x C57B/6, F2 background) were generated as described previously^52^. Mice were divided into two groups AFB1 (Sigma-aldrich) treatment group or control group (treated with tricaprylin, Sigma-aldrich). Mice were intraperitoneally injected with AFB 1 (10 mg per kg of body weight) or solvent at 7 days of age and given equal booster dosages at 2 months. The liver tissues were collected separately from 3-, 6-, 12-, 24-month-old mice in both control group and AFB1 treatment group (n = 3~6). Total RNA was isolated from mouse liver using Trizol Reagent (Invitrogen). C57BL/6JNarl BALB/c and NOD.CB17-Prkdcscid/JNarl mice were purchased from the National Laboratory Animal Center (NLAC), Taipei, Taiwan. Animal study was reviewed and approved by the Institutional Animal Care and Use Committee of Kaohsiung Medical University and performed in compliance with the guidelines.

### Statistical analysis

Data were analyzed using MS excel and Graph Pad Prism 5.0 software. The unpaired two-sided student’s t test was performed to compare groups. The correlation between MYC and GNMT were calculated using Pearson’s correlation coefficient. P < 0.05 considered statistically significant and marked in the figure legends.

## Supplementary information

SUPPLEMENTARY INFORMATION

Supplementary table S1

## Data Availability

The microarray data generated during this study. All data generated or analysed during this study are included in this article (Supplementary Information Table S1) and submitted to the Gene Expression Omnibus (GEO) public database at NCBI (Accession No. GSE75024). http://www.ncbi.nlm.nih.gov/geo/query/acc.cgi?token=axkzwoyojlqbhel&acc=GSE75024.
