# Supplementary material for: Induction of GNMT by 1,2,3,4,6-penta-O-galloyl-beta-D-glucopyranoside through proteasome-independent MYC downregulation in hepatocellular carcinoma
Source: Sci Rep. 2019 Feb 13;9:1968. doi: 10.1038/s41598-018-37292-1 (PMC6374375; doi:10.1038/s41598-018-37292-1)
Supplement: Supplementary file 1 — SUPPLEMENTARY INFORMATION [file 41598_2018_37292_MOESM1_ESM.pdf]

SUPPLEMENTARY INFORMATION

Induction of GNMT by 1,2,3,4,6-penta-O-galloyl-beta-D-glucopyranoside through proteasome-independent MYC downregulation in hepatocellular carcinoma

Rajni Kant[1*], Chia-Hung Yen[1,2,3,4*], Chung-Kuang Lu[5,6], Chien-Yi Tung[7], Pei-Ching Chang[1,8], Jung-Hsien Hung[1,9], Yu-Chang Tyan[1,2,10,11,12‡], Yi-Ming Arthur Chen[1,12,13‡]

[1]Center for Infectious Disease and Cancer Research (CICAR), Kaohsiung Medical University, Kaohsiung, Taiwan

[2]Department of Medical Research, Kaohsiung Medical University Hospital, Kaohsiung, Taiwan

[3]Graduate Institute of Natural Products, College of Pharmacy, Kaohsiung Medical University, Kaohsiung, Taiwan

[4]Research Center for Natural products and Drug Development (CHY), Kaohsiung Medical University, Kaohsiung, Taiwan

[5]National Research Institute of Chinese Medicine, Taipei, Taiwan

[6]Department of Life Sciences and Institute of Genome Sciences, College of Life Science, National Yang-Ming University, Taipei, Taiwan

[7]VYM Genome Research Center, National Yang-Ming University, Taipei, Taiwan

[8]Institute of Microbiology and Immunology, National Yang-Ming University, Taipei, Taiwan

[9]Department and Institute of Pharmacology, National Yang-Ming University, Taipei, Taiwan

[10]Department of Medical Imaging and Radiological Sciences, Kaohsiung Medical University, Kaohsiung, Taiwan

[11]Institute of Medical Science and Technology, National Sun Yat-sen University, Kaohsiung, Taiwan

[12]Graduate Institute of Medicine, College of Medicine, Kaohsiung Medical University, Kaohsiung, Taiwan

[13]Institute of Biomedical Sciences, National Sun Yat-sen University, Kaohsiung, Taiwan

*Equal contributors

‡Corresponding author: Yi-Ming Arthur Chen, No. 100, Shih-Chuan 1st Rd, Kaohsiung City, Taiwan 80708, Phone: 886-7-3117820, Fax: 886-7-3212062, E-mail: arthur@kmu.edu.tw; Yu-Chang Tyan, No. 100, Shih-Chuan 1st Rd, Kaohsiung City, Taiwan 80708, Phone: 886-7-3121101x2357, E-mail: yctyan@kmu.edu.tw

Supplementary figures and legends

Figure S1

a

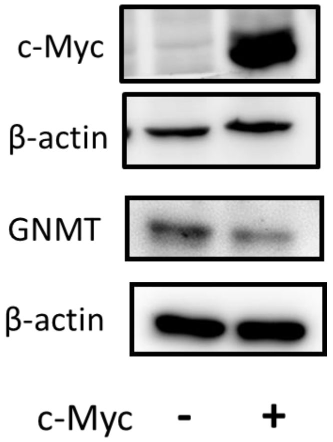

b

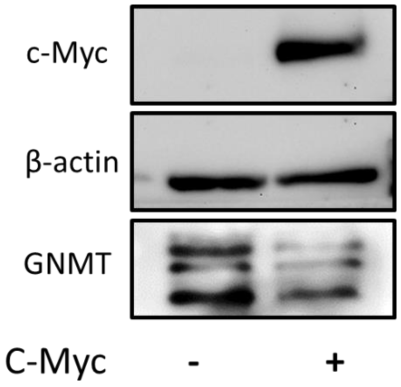

Supplementary Figure S1. Overexpression of MYC inhibits GNMT expression. (a,b) Huh7 (a) and Hep G2 (b) cells were transfected with the pcDNA-MYC or control vector plasmid for 72 hours, then harvested for Protein expression of MYC and GNMT by Immunoblot assay. β-actin expression was used as loading control.

Figure S2

a

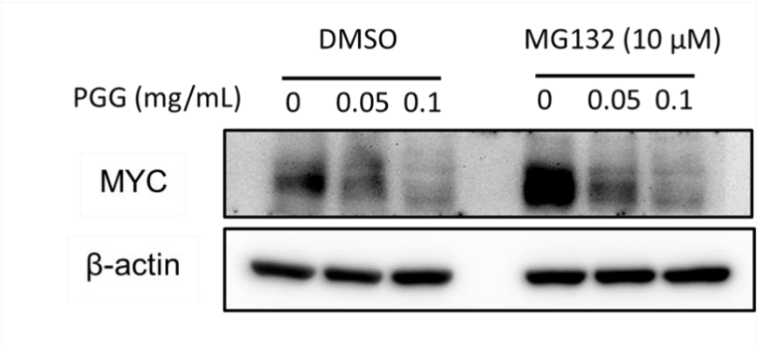

b

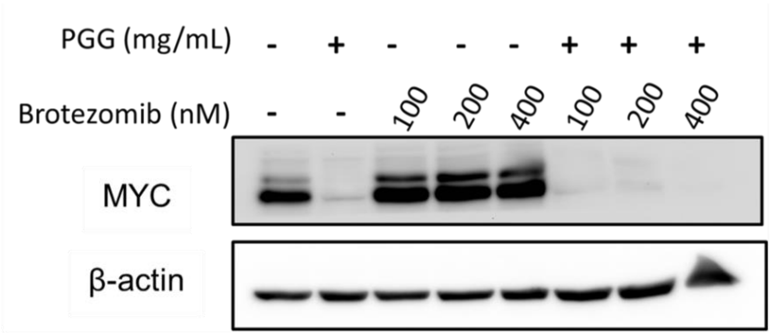

Supplementary Figure S2. PGG promotes the degradation of MYC in a proteasome independent way. (a,b) Huh7 cells were pre-incubated with MG132 **(a)** and velcade **(b)** for 1 hour and then co-treated with PGG (0.1 mg/mL) for 24 hours and subjected to immunoblot analysis for MYC expression. β-actin expression was used as loading control.

Figure S3

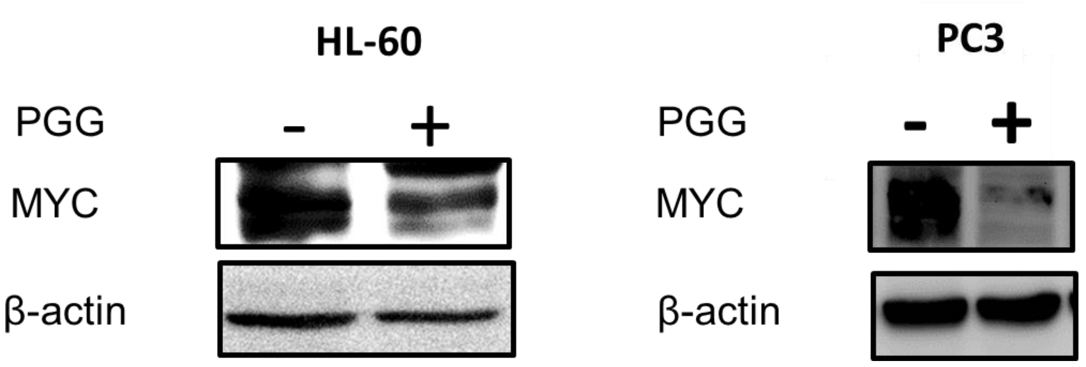

Supplementary Figure S3. PGG inhibits MYC expression in prostate and blood cancer cells. Effect of PGG (0.1 mg/mL) on MYC expression in PC-3 and HL-60 cells were determined by immunoblot analysis after 24 hours of treatment.

Figure S4

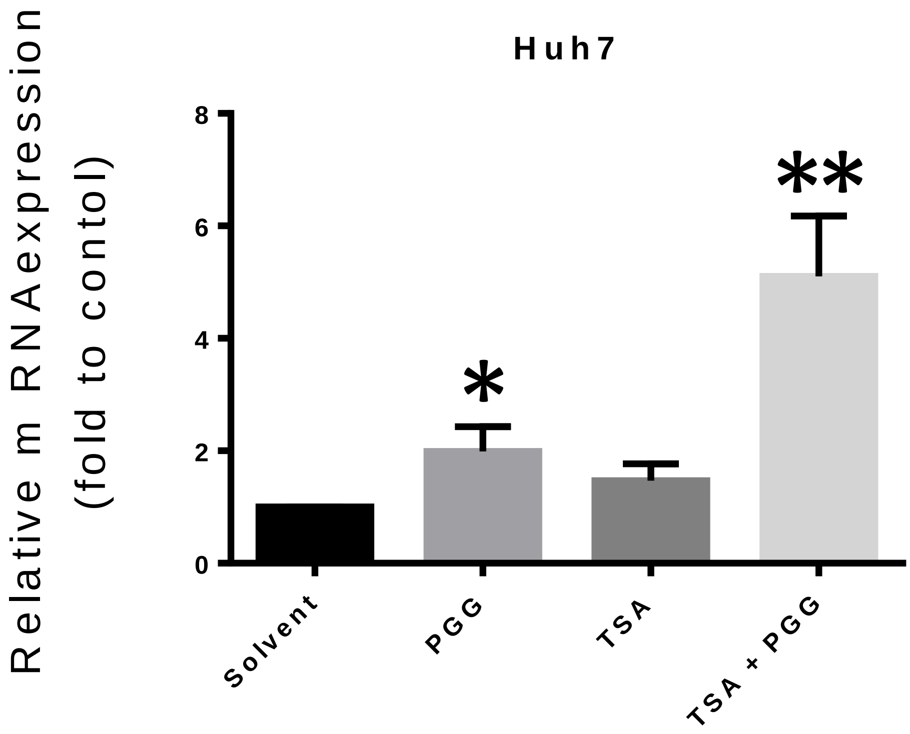

Supplementary Figure S4. Effect of HDAC inhibitor on GNMT mRNA expression. Huh7 cells treated with PGG(0.1 mg/mL) TSA (100nM) and combination for 24 hours. GNMT mRNA level was determined by qRT-PCR. Data was expressed as fold to solvent control. The graph shows the means ± SD (n=3). **P<0.01; *P<0.05 (Student's t-test). TSA(trichostatin A).

The TSA alone increases GNMT mRNA expression dose-dependently published in the previous study[1]. TSA (concentrations >100nM) combination with PGG showed high toxicity to HCC cells and kills most of the cells within 12 hours, therefore, we used the lower concentration of TSA 100nM for this experiment.

Figure S5

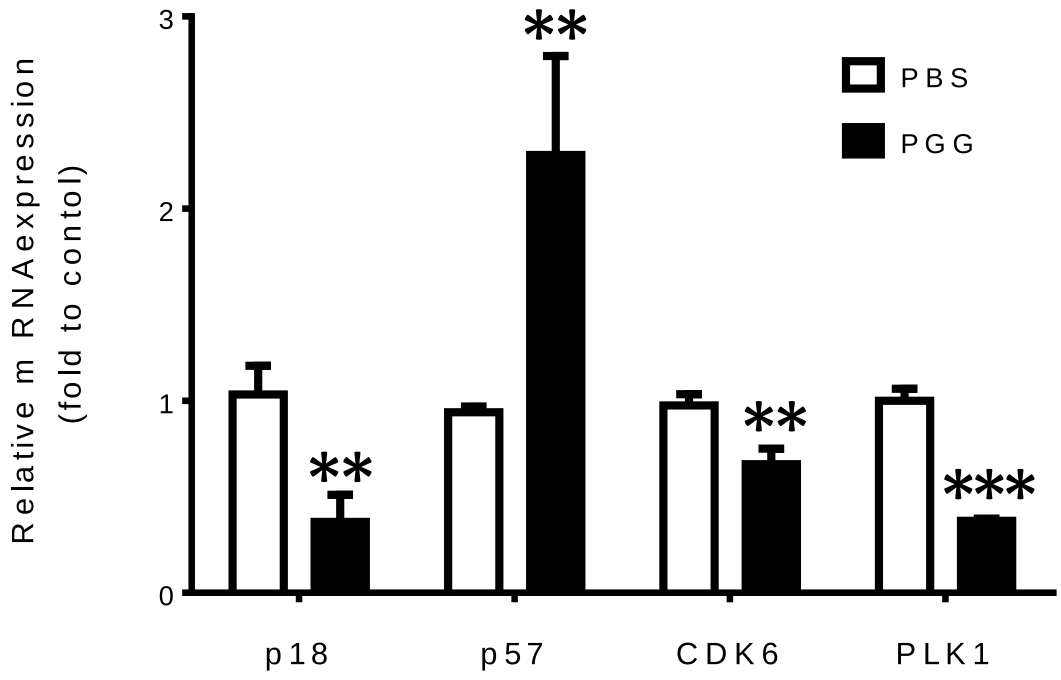

Supplementary Figure S5. Effect of PGG on cell cycle and apoptosis related genes..
Huh7 cells were treated with PGG(0.1 mg/mL) for 24 hours and effect on mRNA expression of indicted genes determined by qRT-PCR. Data was expressed as fold to solvent control. The graph shows the means ± SD (n=3). ***P<0.001; **P<0.01 (Student's t-test).

Figure S6

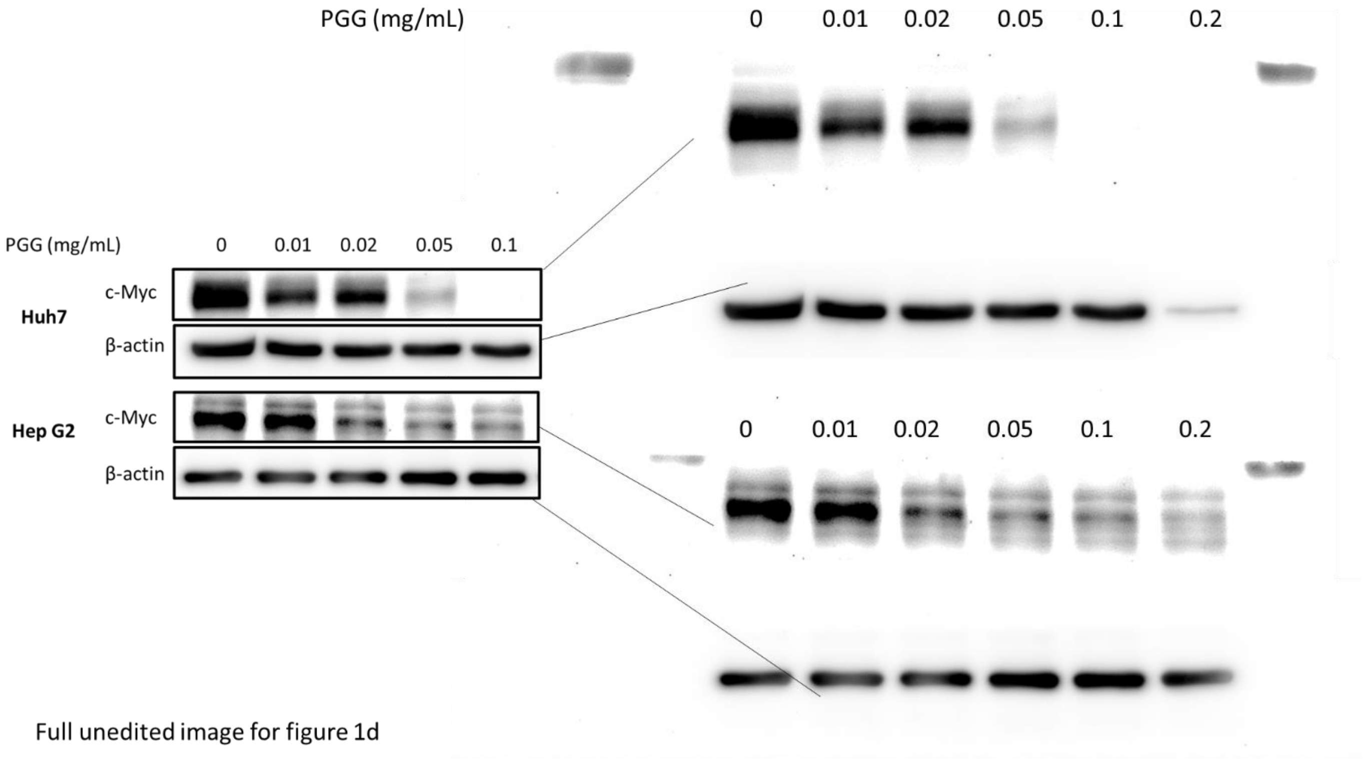

Full unedited image for figure 1d

Figure S7

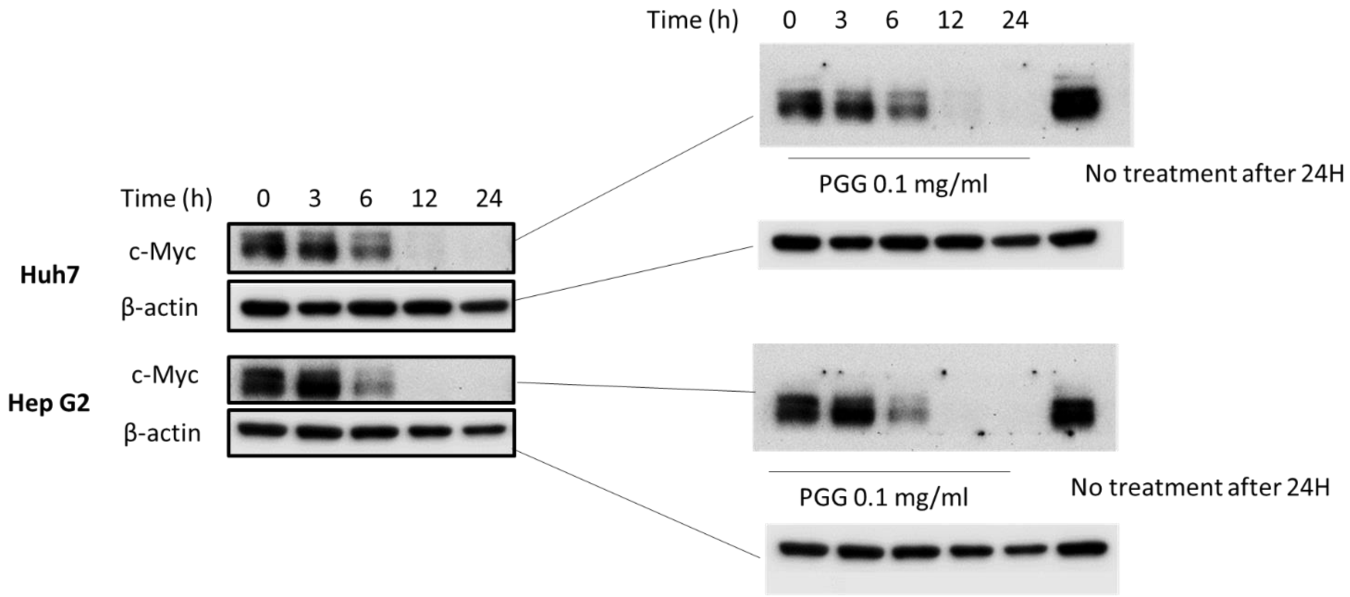

Full unedited image for figure 1e

Figure S8

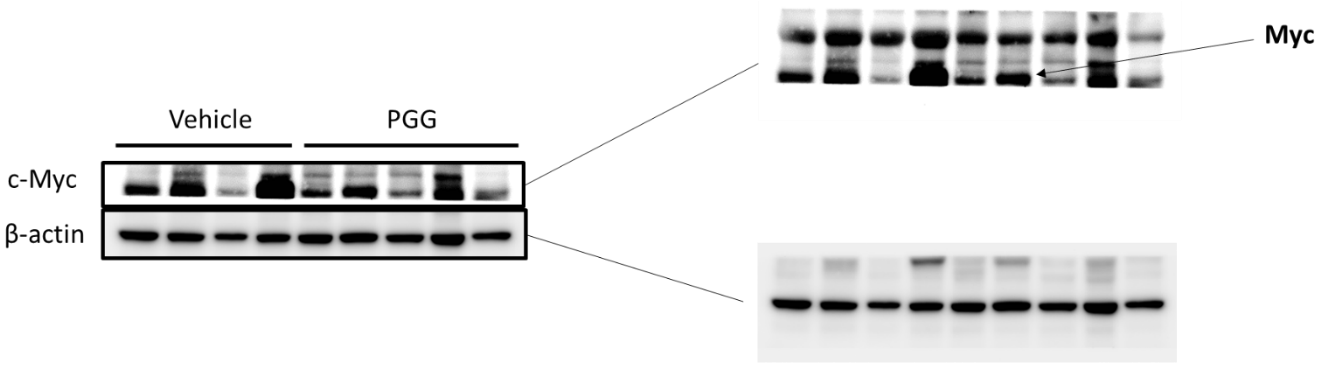

Full unedited image for figure 1h

Figure S9

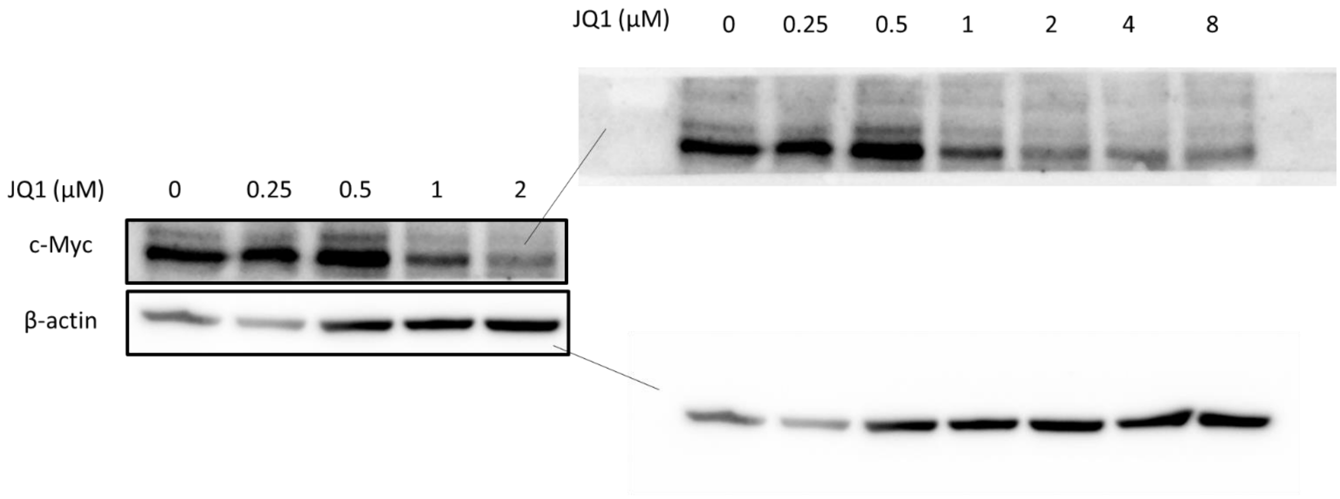

Full unedited image for figure 2f

Figure S10

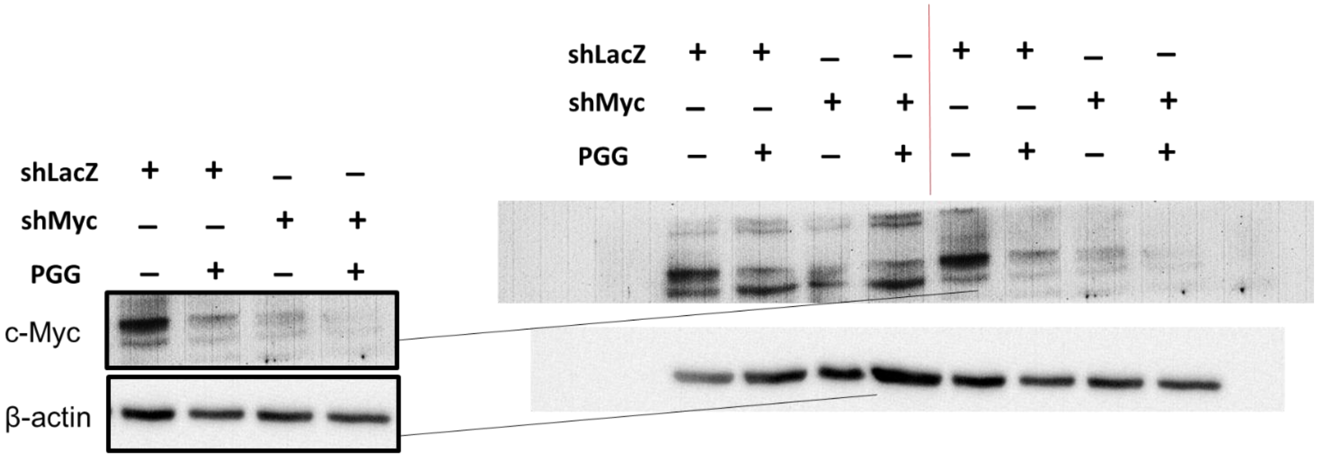

Full unedited image for figure 4b

Figure S11

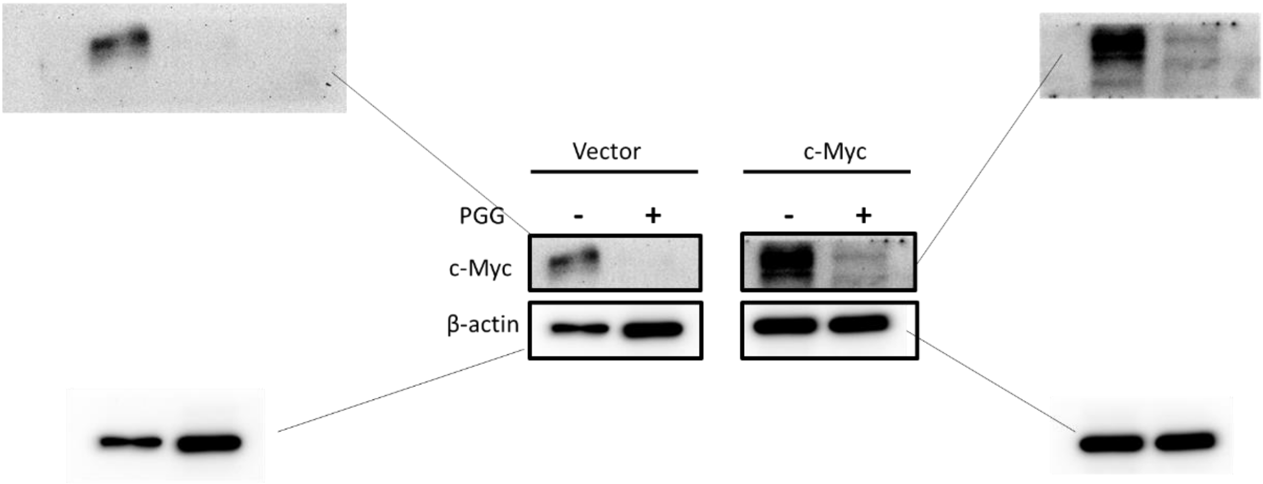

Full unedited image for figure 4e

Figure S12

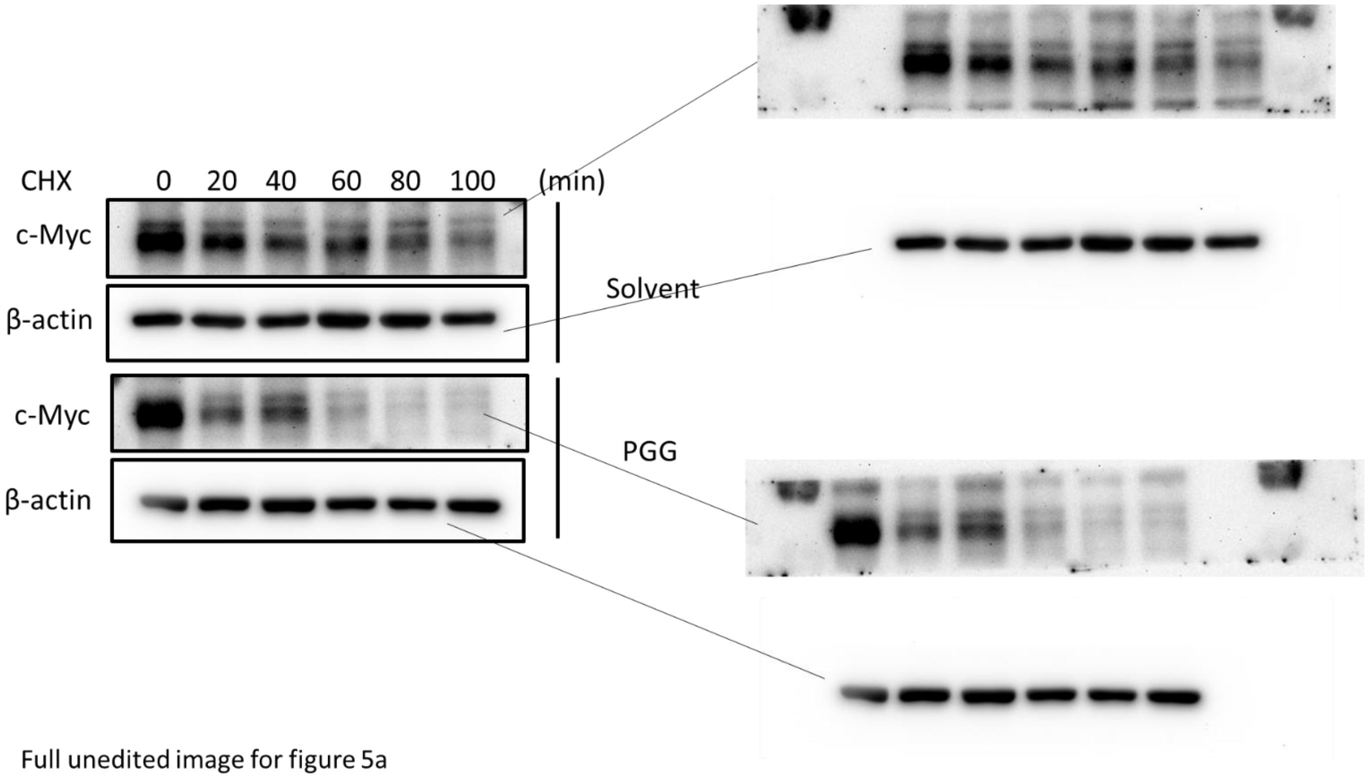

Full unedited image for figure 5a

Figure S13

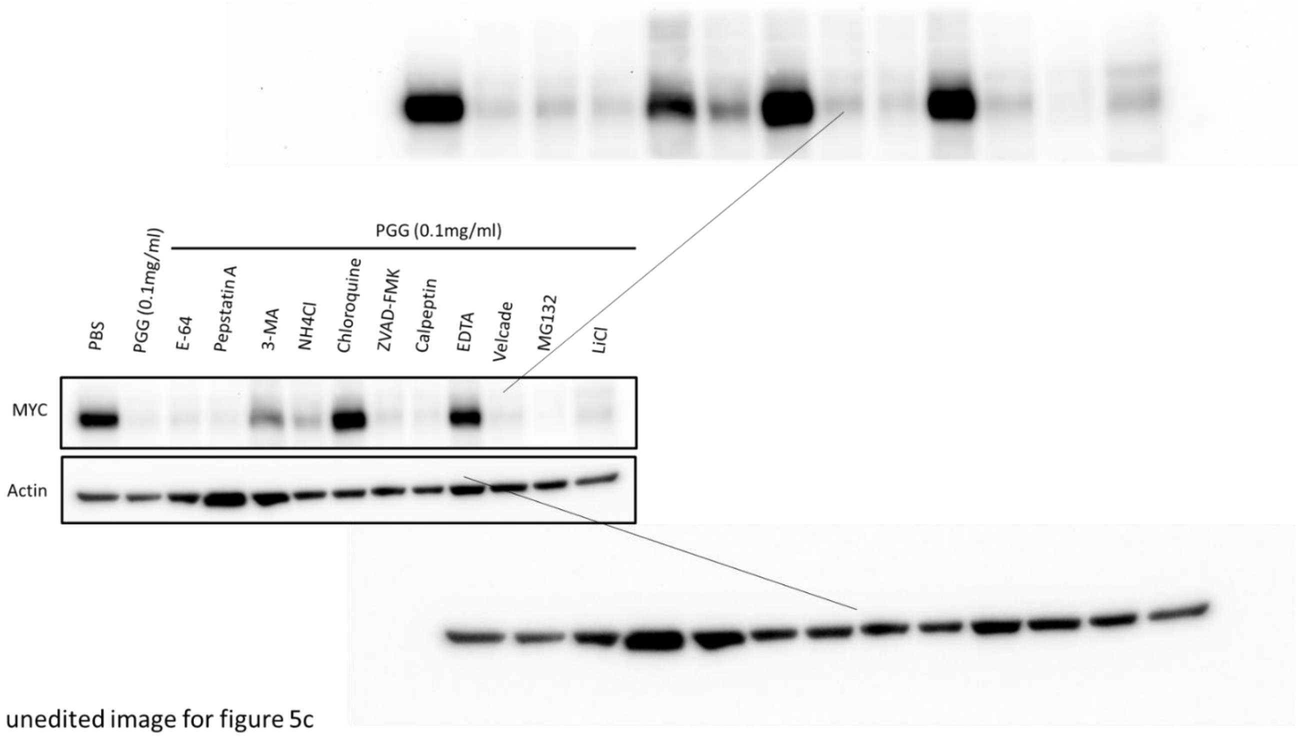

Full unedited image for figure 5c

Supplementary Tables

Supplementary Table S2. ChIP Primers and qPCR Primers

**Name**	**Forward Prime** (5'- to 3')	**Reverse Prime** (5'- to 3')
**Primers for ChIP Binding region**		
GNMT	AAAGGACCTAGCCCAGGA	GATATACAGCTGCCACACG
CCND1	GAAACTTGCACAGGGGTTGT	GCCAAAGAATCTCAGCGACT
**Primers for qPCR**		
GNMT	ACTGGATGACTCTGGACAA	ACTGAGGATGTGGTCGT
MYC	TTCGGTTGTTGCTGATCTGTCT	CCCTCCACTCGGAAGGACTAT
TBP	TGCACAGGAGCCAAGAGTGAA	CACATCACAGCTCCCCACCA
P21	TGAGCCGCGACTGTGATG	GTCTCGGTGACAAAGGTCGAAGTT
P27	TGCAACCGACGATTCTTCTACTCAA	CAAGCAGTGATGTATCTGATAAACAAGGA
Cyclin D1	ACGTCCTGTGCTGCGAAGTGGAAAC	AGTGTTCAATGAAATCGTGCGGGGT
Cyclin D3	CGAGCCTCCTACTTCCAGTG	GGACAGGTAGCGATCCAGGT

1 Kant R, Yen CH, Lu CK, Lin YC, Li JH, Chen YM. Identification of 1,2,3,4,6-Penta-O-galloyl-beta-d-glucopyranoside as a Glycine N-Methyltransferase Enhancer by High-Throughput Screening of Natural Products Inhibits Hepatocellular Carcinoma. *Int J Mol Sci* 2016; 17.
