# Supplementary material for: Induction of GNMT by 1,2,3,4,6-penta-O-galloyl-beta-D-glucopyranoside through proteasome-independent MYC downregulation in hepatocellular carcinoma
Source: Sci Rep. 2019 Feb 13;9:1968. doi: 10.1038/s41598-018-37292-1 (PMC6374375; doi:10.1038/s41598-018-37292-1)
Supplement: Supplementary file 2 — Supplementary table S1 [file 41598_2018_37292_MOESM2_ESM.pdf]

Supplementary Table S1: List of genes up- or down-regulated after PGG treatment in HuH7 cells

Probeset ID	Entrez Gene	Gene Symbol	Gene Title	RefSeq Transcript ID	6h-p-value	6h-qvalue	48h-p-value	48h-qvalue	MeanDiff (6h-PBS)	MeanDiff(48h-PBS)
231202_at	160428	ALDH1L2	aldehyde dehydrogenase 1 family, member L2	NM_001034173 /// NR_027752	1.32E-05	0.000679495	0.000100622	0.000722629	-3.79175	-2.22025
217967_s_at	116496	FAM129A	family with sequence similarity 129, member A	NM_052966	4.35E-05	0.0010699	2.41E-05	0.000380541	-3.69701	-3.29781
208886_at	3005	H1F0	H1 histone family, member 0	NM_005318	3.93E-07	0.000274382	1.11E-05	0.000288032	-3.66812	-1.53845
205302_at	3484	IGFBP1	insulin-like growth factor binding protein 1	NM_000596 /// NM_001013029	5.57E-05	0.00119737	0.00151952	0.00397855	-3.601	1.50193
220491_at	57817	HAMP	hepcidin antimicrobial peptide	NM_021175	9.18E-05	0.00150541	0.000539095	0.00196699	-3.59106	-1.7149
221731_x_at	1462	VCAN	versican	NM_001126336 /// NM_001164097 /// NM_001164098 /// NM_004385	2.34E-06	0.000371918	3.14E-06	0.000207906	-3.54504	-3.60483
204620_s_at	1462	VCAN	versican	NM_001126336 /// NM_001164097 /// NM_001164098 /// NM_004385	2.47E-05	0.000846732	1.95E-05	0.000349517	-3.2758	-3.25519
205767_at	2069	EREG	epiregulin	NM_001432	2.31E-05	0.000826984	1.31E-05	0.000301686	-3.17716	-3.65592
215646_s_at	1462	VCAN	versican	NM_001126336 /// NM_001164097 /// NM_001164098 /// NM_004385	0.000299854	0.00270638	0.000156415	0.000914859	-3.08234	-3.17526
235007_at	55212	BBS7	Bardet-Biedl syndrome 7	NM_018190 /// NM_176824	7.09E-06	0.000532608	3.34E-06	0.000208705	-2.96889	-3.43302
224367_at	84707	BEX2	brain expressed X-linked 2	NM_001168399 /// NM_001168400 /// NM_001168401 /// NM_032621	1.68E-05	0.00074131	0.000130931	0.000825936	-2.68657	1.58457
236321_at	285550	FAM200B	family with sequence similarity 200, member B	NM_001145191	1.72E-05	0.00074501	1.12E-06	0.000157988	-2.66477	-2.93168
211571_s_at	1462	VCAN	versican	NM_001126336 /// NM_001164097 /// NM_001164098 /// NM_004385	0.000365253	0.00302084	0.000508957	0.001901	-2.58372	-2.48696
206354_at	28234	SLCO1B3	solute carrier organic anion transporter family, member 1B3	NM_019844	1.33E-05	0.000680393	9.53E-06	0.000272336	-2.57373	-3.90046
212307_s_at	8473	OGT	O-linked N-acetylglucosamine (GlcNAc) transferase	NM_003605 /// NM_181672 /// NM_181673	9.83E-07	0.000331522	1.30E-06	0.00016106	-2.47333	-2.23208
1568983_a_at	---	---	---	---	1.03E-05	0.000628076	1.63E-05	0.000328625	-2.47182	-2.49206
204619_s_at	1462	VCAN	versican	NM_001126336 /// NM_001164097 /// NM_001164098 /// NM_004385	4.20E-06	0.000458457	5.31E-05	0.000528118	-2.41921	-2.69775
236918_s_at	151827	LRRC34	leucine rich repeat containing 34	NM_001172779 /// NM_001172780 /// NM_153353	3.01E-05	0.000935368	2.32E-05	0.000375129	-2.34204	-3.50785
227982_at	51091	SEPSECS	Sep (O-phosphoserine) tRNA:Sec (selenocysteine) tRNA synthase	NM_001159728 /// NM_016955	6.49E-06	0.000520903	3.82E-05	0.000458309	-2.33317	-1.64177
235516_at	51091	SEPSECS	Sep (O-phosphoserine) tRNA:Sec (selenocysteine) tRNA synthase	NM_001159728 /// NM_016955	4.87E-05	0.00111483	0.00018134	0.000996791	-2.29433	-1.83407
205239_at	374 /// 727738	AREG /// AREGB	amphiregulin /// amphiregulin B	NM_001657 /// XM_001125684	1.77E-05	0.000749535	6.59E-05	0.000582909	-2.21574	-2.40945
222731_at	51201	ZDHHC2	zinc finger, DHHC-type containing 2	NM_016353	3.84E-05	0.00102198	8.09E-05	0.000644459	-2.20697	-1.81137
231979_at	---	---	---	---	0.000511673	0.0035978	0.000672149	0.00227393	-2.1917	-2.30046
236917_at	151827	LRRC34	leucine rich repeat containing 34	NM_001172779 /// NM_001172780 /// NM_153353	0.000816568	0.00474719	0.000450392	0.00175882	-2.16745	-2.89131
214023_x_at	347733	TUBB2B	tubulin, beta 2B class IIb	NM_178012	1.28E-05	0.000676151	4.53E-06	0.000225931	-2.15222	1.996
34031_i_at	889	KRIT1	KRIT1, ankyrin repeat containing	NM_001013406 /// NM_004912 /// NM_194454 /// NM_194455 /// NM_194456	2.55E-06	0.000379211	2.79E-05	0.000401219	-2.12314	-1.66661
204422_s_at	2247	FGF2	fibroblast growth factor 2 (basic)	NM_002006	2.83E-05	0.000905975	0.000472973	0.00181448	-2.11064	-1.86913
226181_at	51175	TUBE1	tubulin, epsilon 1	NM_016262	3.08E-05	0.000936658	0.00025194	0.00122363	-2.10927	-1.97526
235191_at	---	---	---	---	2.67E-06	0.000384712	5.19E-06	0.000236339	-2.07937	-1.5607
209285_s_at	23272	FAM208A	family with sequence similarity 208, member A	NM_001112736 /// NM_015224	1.97E-07	0.000274382	1.89E-07	0.000103238	-2.07503	-2.45424
229377_at	79774	GRTP1	growth hormone regulated TBC protein 1	NM_024719	3.27E-05	0.000961309	4.68E-05	0.000499537	-2.07013	-1.66421
244779_at	51201	ZDHHC2	zinc finger, DHHC-type containing 2	NM_016353	8.99E-05	0.00148895	6.11E-05	0.000559152	-2.05697	-2.22524
207564_x_at	8473	OGT	O-linked N-acetylglucosamine (GlcNAc) transferase	NM_003605 /// NM_181672 /// NM_181673	0.000284527	0.0026368	0.000509394	0.00190149	-2.01291	-1.73176
209672_s_at	54468	MIOS	missing oocyte, meiosis regulator, homolog (Drosophila)	NM_019005	6.66E-05	0.00129906	1.88E-05	0.000344593	-2.00979	-2.4366
225202_at	22836	RHOBTB3	Rho-related BTB domain containing 3	NM_014899	5.78E-06	0.000514161	0.000134545	0.000841192	-2.00942	-1.79421
235274_at	---	---	---	---	8.96E-05	0.00148895	0.000121643	0.000793743	-1.99441	-2.29249
202975_s_at	22836	RHOBTB3	Rho-related BTB domain containing 3	NM_014899	9.89E-06	0.000618405	3.29E-05	0.000427032	-1.99287	-1.88202
203791_at	1657	DMXL1	Dmx-like 1	NM_005509	0.000248269	0.00246413	0.000556099	0.00200691	-1.99027	-1.84426
226670_s_at	80336	PABPC1L	poly(A) binding protein, cytoplasmic 1-like	NM_001124756	0.000300191	0.00270851	0.000555871	0.00200661	-1.94608	-1.7201
230434_at	493911	PHOSPHO2	phosphatase, orphan 2	NM_001008489 /// NM_001199285 /// NM_001199286 /// NM_001199287 /// NM_001199288	1.60E-05	0.0007336	6.62E-06	0.000249228	-1.93687	-2.02353
222156_x_at	100533483 /// 9236	CCPG1 /// DYX1C1-CCPG1	cell cycle progression 1 /// DYX1C1-CCPG1 readthrough (non-protein coding)	NM_001204450 /// NM_001204451 /// NM_004748 /// NM_020739 /// NR_037923	2.70E-07	0.000274382	2.59E-06	0.00019808	-1.92627	-1.60886
202976_s_at	22836	RHOBTB3	Rho-related BTB domain containing 3	NM_014899	1.19E-05	0.000651693	4.08E-05	0.000470536	-1.87459	-1.64779
238877_at	2070	EYA4	eyes absent homolog 4 (Drosophila)	NM_004100 /// NM_172103 /// NM_172104 /// NM_172105	2.73E-05	0.00088594	2.34E-05	0.000375137	-1.86463	-1.72215
203741_s_at	113	ADCY7	adenylate cyclase 7	NM_001114	9.60E-05	0.00153591	0.000104768	0.000736925	-1.85041	-1.67674
225699_at	285958	SNHG15	small nucleolar RNA host gene 15 (non-protein coding)	NR_003697	1.18E-05	0.000651423	5.63E-06	0.000239435	-1.84843	-2.13997
212816_s_at	875	CBS	cystathionine-beta-synthase	NM_000071 /// NM_001178008 /// NM_001178009	6.85E-06	0.000530552	8.86E-06	0.000263784	-1.84659	-1.93576
236261_at	114880	OSBPL6	oxysterol binding protein-like 6	NM_001201480 /// NM_001201481 /// NM_001201482 /// NM_032523 /// NM_145739	3.15E-07	0.000274382	7.09E-05	0.000603808	-1.83272	-2.03663
212486_s_at	2534	FYN	FYN oncogene related to SRC, FGR, YES	NM_001242779 /// NM_002037 /// NM_153047 /// NM_153048	0.000102778	0.00159588	4.02E-05	0.000468433	-1.83089	-2.14399
214651_s_at	00534589 /// 3205 /// 44292	XA10-HOXA9 /// HOXA9 /// MIR1	HOXA10-HOXA9 readthrough /// homeobox A9 /// microRNA 196b	NM_152739 /// NR_029911 /// NR_037940	3.77E-05	0.00101441	3.05E-05	0.000416123	-1.78276	-2.12246
202431_s_at	4609	**MYC**	v-myc myelocytomatosis viral oncogene homolog (avian)	NM_002467	1.61E-05	0.0007336	8.01E-05	0.000642137	-1.78253	-1.85594
204742_s_at	23047	PDS5B	PDS5, regulator of cohesion maintenance, homolog B (S. cerevisiae)	NM_015032 /// NM_015928	7.35E-08	0.000220355	1.62E-06	0.000175687	-1.77885	-2.79034
238575_at	114880	OSBPL6	oxysterol binding protein-like 6	NM_001201480 /// NM_001201481 /// NM_001201482 /// NM_032523 /// NM_145739	0.00014512	0.0018649	0.000271289	0.00127767	-1.76824	-1.85224
1553972_a_at	875	CBS	cystathionine-beta-synthase	NM_000071 /// NM_001178008 /// NM_001178009	1.37E-05	0.00068789	2.16E-05	0.000365823	-1.76348	-1.91854
206525_at	2569	GABRR1	gamma-aminobutyric acid (GABA) A receptor, rho 1	NM_001256703 /// NM_001256704 /// NM_001267582 /// NM_002042	0.000178814	0.0020791	1.43E-05	0.000312495	-1.75878	-2.51479
207308_at	6579	SLCO1A2	solute carrier organic anion transporter family, member 1A2	NM_005075 /// NM_021094 /// NM_134431	0.000120956	0.00171309	7.26E-06	0.000254296	-1.74375	-2.28731
1562099_at	---	---	---	---	8.90E-06	0.000589169	7.32E-06	0.000254296	-1.73292	-1.78618
231269_at	10973	ASCC3	activating signal cointegrator 1 complex subunit 3	NM_006828 /// NM_022091	1.40E-06	0.000331522	5.97E-07	0.000128876	-1.73075	-1.51587
206314_at	55888	ZNF167	zinc finger protein 167	NM_018651 /// NM_025169	2.64E-07	0.000274382	0.000212693	0.00109795	-1.71976	-2.09836
238599_at	134728	IRAK1BP1	interleukin-1 receptor-associated kinase 1 binding protein 1	NM_001010844	0.000322092	0.00281642	6.92E-05	0.000596446	-1.71745	-2.52184
221539_at	1978	EIF4EBP1	eukaryotic translation initiation factor 4E binding protein 1	NM_004095	6.37E-06	0.000520519	2.31E-06	0.000189358	-1.70705	-1.51223
233341_s_at	84172	POLR1B	polymerase (RNA) I polypeptide B, 128kDa	NM_001137604 /// NM_019014	4.93E-06	0.000481555	2.18E-07	0.000103238	-1.69366	-1.50051
209292_at	3400	ID4	inhibitor of DNA binding 4, dominant negative helix-loop-helix protein	NM_001546	5.80E-06	0.000514161	5.32E-07	0.000126065	-1.68915	-3.26405
217966_s_at	116496	FAM129A	family with sequence similarity 129, member A	NM_052966	0.000537675	0.00369871	0.000532683	0.00195219	-1.68311	-1.50139
238681_at	284161	GDPD1	glycerophosphodiester phosphodiesterase domain containing 1	NM_001165993 /// NM_001165994 /// NM_182569	4.31E-06	0.00046339	1.25E-05	0.000299017	-1.66812	-2.48125
242136_x_at	403340	MGC70870	C-terminal binding protein 2 pseudogene	NR_003682	0.000395451	0.00313787	0.000291235	0.00133775	-1.66458	-1.56765
228661_s_at	100506029 /// 100506051	LOC100506029 /// LOC100506051	uncharacterized LOC100506029 /// uncharacterized LOC100506051	XR_110481 /// XR_110482 /// XR_112086 /// XR_133246	7.73E-06	0.000549234	3.06E-05	0.000416123	-1.66358	-2.69617
1554067_at	144577	C12orf66	chromosome 12 open reading frame 66	NM_152440	3.50E-05	0.000990344	0.000882091	0.00273897	-1.65719	-2.30737
1556194_a_at	---	---	---	---	5.43E-05	0.00118519	2.20E-05	0.000367817	-1.65505	-2.0889
241933_at	55278	QRSL1	glutaminyl-tRNA synthase (glutamine-hydrolyzing)-like 1	NM_018292	0.000140318	0.00183198	0.000144793	0.000879118	-1.63759	-1.80001
213954_at	26049	FAM169A	family with sequence similarity 169, member A	NM_015566 /// NR_046462	0.000159347	0.00196358	0.000200731	0.00106095	-1.63382	-2.38597
220892_s_at	29968	PSAT1	phosphoserine aminotransferase 1	NM_021154 /// NM_058179	0.00047739	0.00347364	0.000716495	0.00237211	-1.63158	-1.59051
1553689_s_at	131965	METTL6	methyltransferase like 6	NM_152396	1.75E-06	0.000347641	8.81E-09	6.53E-05	-1.6292	-2.29616
209349_at	10111	RAD50	RAD50 homolog (S. cerevisiae)	NM_005732 /// NM_133482	1.41E-05	0.000697032	9.89E-06	0.000276759	-1.62246	-1.56599
209905_at	100534589 /// 3205	HOXA10-HOXA9 /// HOXA9	HOXA10-HOXA9 readthrough /// homeobox A9	NM_152739 /// NR_037940	4.70E-05	0.00109887	1.98E-05	0.000351271	-1.62006	-1.6351
223167_s_at	29761	USP25	ubiquitin specific peptidase 25	NM_013396	1.46E-05	0.000705975	2.76E-05	0.00039954	-1.61477	-2.30704
207300_s_at	2155	F7	coagulation factor VII (serum prothrombin conversion accelerator)	NM_000131 /// NM_001267554 /// NM_019616 /// NR_051961	8.22E-05	0.00143722	4.71E-05	0.000500898	-1.61205	-1.52148
217997_at	22822	PHLDA1	pleckstrin homology-like domain, family A, member 1	NM_007350	2.44E-07	0.000274382	7.32E-08	0.000103238	-1.61128	-4.04801
235763_at	204962	SLC44A5	solute carrier family 44, member 5	NM_001130058 /// NM_152697	6.72E-05	0.00130444	1.53E-05	0.000318737	-1.60738	-1.94976
219291_at	56986	DTWD1	DTW domain containing 1	NM_001144955 /// NM_020234	8.42E-05	0.0014538	5.42E-05	0.000531952	-1.60708	-1.99727
227559_at	29078	NDUFAF4	NADH dehydrogenase (ubiquinone) complex I, assembly factor 4	NM_014165	1.89E-05	0.000768225	1.04E-05	0.000281936	-1.60011	-3.49088
223805_at	114880	OSBPL6	oxysterol binding protein-like 6	NM_001201480 /// NM_001201481 /// NM_001201482 /// NM_032523 /// NM_145739	7.83E-05	0.00141181	0.00016126	0.000930217	-1.58966	-1.57702

236994_at	---	---	---	---	0.00014108	0.00183825	8.46E-05	0.000659882	**-1.58453**	**-1.83042**
227241_at	143662	MUC15	mucin 15, cell surface associated	NM_001135091 /// NM_001135092 /// NM_145650	0.000179598	0.00208104	8.32E-05	0.000653498	**-1.58218**	**-2.27138**
209602_s_at	2625	GATA3	GATA binding protein 3	NM_001002295 /// NM_002051	9.70E-05	0.00154603	6.83E-05	0.000593463	**-1.55677**	**-2.13967**
236665_at	343099	CCDC18	coiled-coil domain containing 18	NM_206886	0.000602176	0.00395298	0.00069582	0.00232533	**-1.55353**	**-1.56847**
243065_at	---	---	---	---	0.000691845	0.00430455	0.00148061	0.00390912	**-1.54604**	**-1.64629**
228573_at	118429	ANTXR2	anthrax toxin receptor 2	NM_001145794 /// NM_058172	6.57E-05	0.00129218	7.65E-05	0.000629659	**-1.54226**	**-2.14436**
204853_at	4999	**ORC2**	origin recognition complex, subunit 2	NM_006190 /// NR_033915	5.10E-05	0.00114124	6.60E-06	0.000249228	**-1.53989**	**-1.69616**
227270_at	285550	FAM200B	family with sequence similarity 200, member B	NM_001145191	0.000234664	0.00239335	8.58E-06	0.000261476	**-1.53873**	**-2.1039**
225424_at	57678	GPAM	glycerol-3-phosphate acyltransferase, mitochondrial	NM_001244949 /// NM_020918	0.000363917	0.00301613	0.000176929	0.000981614	**-1.53856**	**-1.91941**
229178_at	283659	PRTG	protogenin	NM_173814	0.000205191	0.00223018	2.32E-05	0.000375129	**-1.53666**	**-3.15747**
210662_at	8942	KYNU	kynureninase	NM_001032998 /// NM_001199241 /// NM_003937	1.72E-05	0.00074501	1.53E-05	0.000318737	**-1.53422**	**-1.89171**
205046_at	1062	**CENPE**	centromere protein E, 312kDa	NM_001813	1.87E-06	0.000362042	9.00E-06	0.000264916	**-1.53117**	**-1.86457**
220205_at	7179	TPTE	transmembrane phosphatase with tensin homology	NM_199259 /// NM_199260 /// NM_199261	0.000182163	0.00209885	0.000175709	0.000978265	**-1.52925**	**-1.68172**
1568807_a_at	100505538	RBM26-AS1	RBM26 antisense RNA 1 (non-protein coding)	NR_038991	0.000141207	0.00183825	0.00117788	0.00334386	**-1.5021**	**-1.57957**
210587_at	83729	INHBE	inhibin, beta E	NM_031479	7.51E-06	0.000548089	3.94E-06	0.00021731	**-1.55533**	**1.52013**
219803_at	27329	ANGPTL3	angiopoietin-like 3	NM_014495	0.000659228	0.00417516	0.000214788	0.00110364	**1.52828**	**-1.85803**
202238_s_at	4837	NNMT	nicotinamide N-methyltransferase	NM_006169	0.000383233	0.00309383	0.00036336	0.00153846	**1.51465**	**1.57719**
224314_s_at	54583	EGLN1	egl nine homolog 1 (C. elegans)	NM_022051	6.02E-06	0.000515252	3.95E-05	0.000464144	**1.51636**	**2.23959**
224833_at	2113	ETS1	v-ets erythroblastosis virus E26 oncogene homolog 1 (avian)	NM_001143820 /// NM_001162422 /// NM_005238	0.000220275	0.00231303	1.16E-05	0.00029282	**1.5203**	**3.45399**
202769_at	901	**CCNG2**	cyclin G2	NM_004354	3.58E-06	0.00042511	2.69E-07	0.000105892	**1.52292**	**3.23533**
226347_at	170384	FUT11	fucosyltransferase 11 (alpha (1,3) fucosyltransferase)	NM_173540	0.000148697	0.00188301	2.42E-05	0.000380825	**1.54539**	**2.72747**
209007_s_at	57035	C1orf63	chromosome 1 open reading frame 63	NM_020317 /// NM_207035	0.000550391	0.00374892	6.87E-05	0.000594419	**1.55856**	**2.79882**
202972_s_at	10144	FAM13A	family with sequence similarity 13, member A	NM_001015045 /// NM_001265578 /// NM_001265579 /// NM_001265580 /// NM_014883	0.000558482	0.00378005	0.000357798	0.00152538	**1.56377**	**2.1765**
231579_s_at	7077	TIMP2	TIMP metallopeptidase inhibitor 2	NM_003255	5.96E-06	0.000515252	4.05E-07	0.000120179	**1.57258**	**2.79475**
202464_s_at	5209	PFKFB3	6-phosphofructo-2-kinase/fructose-2,6-biphosphatase 3	NM_001145443 /// NM_004566	8.85E-06	0.000588031	2.53E-07	0.000105892	**1.57702**	**1.79996**
224560_at	7077	TIMP2	TIMP metallopeptidase inhibitor 2	NM_003255	0.000221313	0.00232212	2.08E-05	0.000361244	**1.59655**	**2.88906**
202998_s_at	4017	LOXL2	lysyl oxidase-like 2	NM_002318	3.79E-05	0.00101544	6.53E-07	0.000132509	**1.60577**	**4.31487**
202375_at	9871	SEC24D	SEC24 family, member D (S. cerevisiae)	NM_014822	0.000555373	0.0037711	0.000148202	0.000887853	**1.61245**	**1.76636**
243982_at	---	---	---	---	0.000129837	0.00176874	0.000291022	0.00133716	**1.62724**	**1.7899**
202237_at	4837	NNMT	nicotinamide N-methyltransferase	NM_006169	0.000281284	0.00262374	0.000193076	0.00103427	**1.63097**	**1.81126**
211126_s_at	1466	CSRP2	cysteine and glycine-rich protein 2	NM_001321	8.98E-06	0.000589169	3.84E-05	0.000459959	**1.6342**	**1.55965**
236480_at	100506211	MIR210HG	MIR210 host gene (non-protein coding)	NR_038262	0.000154756	0.00192992	0.000163753	0.000940932	**1.6388**	**1.64724**
202770_s_at	901	**CCNG2**	cyclin G2	NM_004354	1.57E-05	0.000732432	8.14E-07	0.000142405	**1.65836**	**3.33112**
202912_at	133	ADM	adrenomedullin	NM_001124	1.02E-06	0.000331522	1.18E-07	0.000103238	**1.67845**	**2.24861**
243296_at	10135	NAMPT	Nicotinamide phosphoribosyltransferase	NM_005746 /// NM_182790	0.000207996	0.00224611	8.46E-05	0.000659882	**1.67948**	**2.2394**
211559_s_at	901	**CCNG2**	cyclin G2	NM_004354	0.000309367	0.00274812	1.43E-05	0.000312543	**1.68275**	**3.32983**
202973_x_at	10144	FAM13A	family with sequence similarity 13, member A	NM_001015045 /// NM_001265578 /// NM_001265579 /// NM_001265580 /// NM_014883	3.07E-05	0.000936658	1.26E-05	0.000299017	**1.69181**	**2.35145**
228648_at	116844	LRG1	leucine-rich alpha-2-glycoprotein 1	NM_052972	4.21E-05	0.00105497	3.25E-06	0.000208705	**1.69546**	**2.30597**
208789_at	284119	PTRF	polymerase I and transcript release factor	NM_012232	0.000537619	0.00369871	0.000622757	0.00216163	**1.76091**	**1.69146**
226348_at	---	---	---	---	3.17E-06	0.000407194	1.11E-05	0.000288032	**1.76996**	**2.84707**
228953_at	123720	WHAMM	WAS protein homolog associated with actin, golgi membranes and microtubules	NM_001080435	1.03E-06	0.000331522	1.15E-07	0.000103238	**1.77508**	**3.63893**
242996_at	9617	MTRF1	mitochondrial translational release factor 1	NM_004294	0.000440158	0.00332573	0.000704451	0.00234275	**1.78721**	**2.19798**
231121_at	84343	HPS3	Hermansky-Pudlak syndrome 3	NM_032383	5.50E-06	0.000507294	2.11E-06	0.000186913	**1.80475**	**2.08429**
217047_s_at	10144	FAM13A	family with sequence similarity 13, member A	NM_001015045 /// NM_001265578 /// NM_001265579 /// NM_001265580 /// NM_014883	3.24E-05	0.000957446	1.29E-05	0.00029992	**1.81619**	**2.40927**
242457_at	---	---	---	---	1.11E-05	0.000637317	2.38E-05	0.000377758	**1.82344**	**1.78354**
215209_at	9871	SEC24D	SEC24 family, member D (S. cerevisiae)	NM_014822	1.50E-05	0.00071087	6.95E-05	0.00059815	**1.84393**	**2.41531**
244841_at	10802	SEC24A	SEC24 family, member A (S. cerevisiae)	NM_001252231 /// NM_021982	7.98E-07	0.000329091	2.81E-06	0.000204457	**1.8482**	**4.49734**
207030_s_at	1466	CSRP2	cysteine and glycine-rich protein 2	NM_001321	2.02E-06	0.000362042	6.97E-06	0.000252333	**1.86782**	**1.80775**
241262_at	---	---	---	---	0.000147896	0.00187734	9.14E-05	0.000687736	**1.87804**	**2.28085**
243020_at	---	---	---	---	2.18E-05	0.000810078	8.23E-06	0.000260132	**1.90022**	**2.95082**
219014_at	51316	PLAC8	placenta-specific 8	NM_001130715 /// NM_001130716 /// NM_016619	6.59E-06	0.000524324	7.97E-06	0.000258347	**1.925**	**2.61721**
1560622_at	---	---	---	---	1.29E-05	0.000679483	1.12E-06	0.000157988	**1.94838**	**1.94332**
209949_at	4688	NCF2	neutrophil cytosolic factor 2	NM_000433 /// NM_001127651 /// NM_001190789 /// NM_001190794	2.04E-06	0.000362042	1.08E-06	0.000156316	**1.96058**	**2.38408**
222316_at	---	---	---	---	1.94E-05	0.000770538	0.000145609	0.000881832	**1.9644**	**1.64372**
200632_s_at	10397	NDRG1	N-myc downstream regulated 1	NM_001135242 /// NM_001258432 /// NM_001258433 /// NM_006096	0.000121308	0.00171309	1.72E-05	0.000338712	**1.96451**	**3.65714**
238551_at	170384	FUT11	fucosyltransferase 11 (alpha (1,3) fucosyltransferase)	NM_173540	3.69E-05	0.001009	2.09E-05	0.000361371	**1.96859**	**2.33681**
210538_s_at	330	BIRC3	baculoviral IAP repeat containing 3	NM_001165 /// NM_182962	7.70E-05	0.0013975	8.11E-06	0.000259362	**2.01162**	**3.58341**
218717_s_at	55214	LEPREL1	leprecan-like 1	NM_001134418 /// NM_018192	8.84E-07	0.000329091	2.01E-06	0.00018478	**2.03316**	**2.32227**
232628_at	---	---	---	---	2.04E-05	0.000782538	4.61E-06	0.000225937	**2.0534**	**2.86414**
204304_s_at	8842	PROM1	prominin 1	NM_001145847 /// NM_001145848 /// NM_001145849 /// NM_001145850 /// NM_001145851 /// NM	1.15E-05	0.000645941	4.40E-06	0.000223841	**2.0658**	**3.63843**
206457_s_at	1733	DIO1	deiodinase, iodothyronine, type I	NM_000792 /// NM_001039715 /// NM_001039716 /// NM_213593	6.15E-05	0.00124799	1.80E-06	0.000180854	**2.076**	**2.17079**
229540_at	3516	RBPJ	recombination signal binding protein for immunoglobulin kappa J region	NM_005349 /// NM_015874 /// NM_203283 /// NM_203284	1.04E-06	0.000331522	1.30E-06	0.00016106	**2.07842**	**2.69378**
205780_at	638	BIK	BCL2-interacting killer (apoptosis-inducing)	NM_001197	0.00021118	0.00225828	0.000172404	0.000971548	**2.08024**	**2.34228**
1555476_at	3658	IREB2	iron-responsive element binding protein 2	NM_004136	8.87E-05	0.0014864	2.26E-05	0.000372058	**2.18577**	**2.06537**
243927_x_at	25962	KIAA1429	KIAA1429	NM_015496 /// NM_183009	0.00019743	0.00218719	0.000491216	0.00185885	**2.19295**	**2.15013**
215855_s_at	7110	TMF1	TATA element modulatory factor 1	NM_007114	0.000105053	0.00160534	3.58E-05	0.000444157	**2.21319**	**2.83794**
201650_at	3880	KRT19	keratin 19	NM_002276	0.000182028	0.00209839	0.000274422	0.00128672	**2.22307**	**2.02543**
205034_at	9134	**CCNE2**	cyclin E2	NM_004702 /// NM_057735 /// NM_057749	0.000113424	0.00166613	0.000265369	0.00126102	**2.26089**	**1.74277**
203632_s_at	51704	GPRC5B	G protein-coupled receptor, family C, group 5, member B	NM_016235	0.000161617	0.00197937	0.000137918	0.000852919	**2.26341**	**2.43904**
1552485_at	114294	LACTB	lactamase, beta	NM_032857 /// NM_171846	3.79E-05	0.00101523	0.000124047	0.000802427	**2.31534**	**1.94788**
236561_at	7046	TGFBR1	transforming growth factor, beta receptor 1	NM_001130916 /// NM_004612	0.00012981	0.00176874	5.36E-05	0.000529448	**2.35252**	**3.02881**
213349_at	23023	TMCC1	transmembrane and coiled-coil domain family 1	NM_001017395 /// NM_001128224 /// NM_015008 /// NR_033361	0.000740426	0.00449379	0.000519331	0.00192165	**2.37655**	**2.61006**
236829_at	---	---	---	---	2.97E-05	0.000930161	8.70E-06	0.000261902	**2.49874**	**3.16296**
242243_at	7110	TMF1	TATA element modulatory factor 1	NM_007114	4.55E-05	0.00108389	0.000182901	0.00100125	**2.50947**	**3.17511**
241905_at	5286	PIK3C2A	Phosphoinositide-3-kinase, class 2, alpha polypeptide	NM_002645	2.15E-05	0.000806462	3.29E-05	0.000427032	**2.51365**	**3.55338**
205220_at	8843	HCAR3	hydroxycarboxylic acid receptor 3	NM_006018	1.97E-05	0.000773002	4.20E-06	0.000221146	**2.51862**	**3.66438**
210517_s_at	9590	AKAP12	A kinase (PRKA) anchor protein 12	NM_005100 /// NM_144497	1.40E-07	0.000274382	2.98E-07	0.000105892	**2.53449**	**3.31651**
227926_s_at	---	---	---	---	1.89E-05	0.000768225	0.000736577	0.00241502	**2.63069**	**1.82161**
242907_at	2634	GBP2	guanylate binding protein 2, interferon-inducible	NM_004120	0.000124974	0.00173847	2.21E-05	0.000368583	**2.64941**	**3.18227**
201798_s_at	26509	MYOF	myoferlin	NM_013451 /// NM_133337	3.40E-05	0.000974559	3.77E-05	0.000455189	**2.69439**	**2.67266**
228966_at	80025	PANK2	Pantothenate kinase 2	NM_024960 /// NM_153637 /// NM_153638 /// NM_153639 /// NM_153640 /// NM_153641	3.02E-05	0.000935368	9.13E-05	0.00068716	**2.86327**	**2.89368**
204159_at	1031	CDKN2C	cyclin-dependent kinase inhibitor 2C (p18, inhibits CDK4)	NM_001262 /// NM_078626	5.95E-05	0.00123187	0.000177772	0.000984324	**3.05517**	**2.21637**
228499_at	5210	PFKFB4	6-phosphofructo-2-kinase/fructose-2,6-biphosphatase 4	NM_004567	6.89E-07	0.000320752	6.35E-08	0.000103238	**3.38903**	**4.53776**
220987_s_at	56672 /// 81788	AKIP1 /// NUAK2	A kinase (PRKA) interacting protein 1 /// NUAK family, SNF1-like kinase, 2	NM_001206645 /// NM_001206646 /// NM_001206647 /// NM_001206648 /// NM_020642 /// NM_03	5.97E-06	0.000515252	6.30E-06	0.00024863	**3.41583**	**3.52562**

217546_at	4499	MT1M	metallothionein 1M	NM_176870	4.66E-06	0.000475241	8.40E-05	0.000658266	**3.44555**	**1.65573**
205547_s_at	6876	TAGLN	transgelin	NM_001001522 /// NM_003186	2.47E-05	0.000846732	0.000781254	0.00251537	**3.50227**	**1.52085**
234040_at	3070	HELLS	helicase, lymphoid-specific	NM_018063	6.99E-05	0.00132802	0.000210294	0.00109176	**4.55668**	**3.38842**
